# A standardised hERG phenotyping pipeline to evaluate *KCNH2* genetic variant pathogenicity

**DOI:** 10.1002/ctm2.609

**Published:** 2021-11-23

**Authors:** Barbara Oliveira‐Mendes, Sylvain Feliciangeli, Mélissa Ménard, Frank Chatelain, Malak Alameh, Jérôme Montnach, Sébastien Nicolas, Béatrice Ollivier, Julien Barc, Isabelle Baró, Jean‐Jacques Schott, Vincent Probst, Florence Kyndt, Isabelle Denjoy, Florian Lesage, Gildas Loussouarn, Michel De Waard

**Affiliations:** ^1^ l'Institut du Thorax Inserm UMR 1087/CNRS UMR 6291 Nantes France; ^2^ Labex ICST, Université Côte d'Azur, INSERM Centre National de la Recherche Scientifique, Institut de Pharmacologie Moléculaire et Cellulaire Valbonne France; ^3^ CHU Nantes, l'Institut du Thorax, INSERM, CNRS UNIV Nantes Nantes France; ^4^ Service de Cardiologie et CNMR Maladies Cardiaques Héréditaires Rares Hôpital Bichat Paris France

**Keywords:** arrhythmias, diagnostic testing, genetic variant, hERG ion channel, pathogenicity, QT syndrome, translational medicine

## Abstract

**Background and aims:**

Mutations in *KCNH2* cause long or short QT syndromes (LQTS or SQTS) predisposing to life‐threatening arrhythmias. Over 1000 hERG variants have been described by clinicians, but most remain to be characterised. The objective is to standardise and accelerate the phenotyping process to contribute to clinician diagnosis and patient counselling. *In silico* evaluation was also included to characterise the structural impact of the variants.

**Methods:**

We selected 11 variants from known LQTS patients and two variants for which diagnosis was problematic. Using the Gibson assembly strategy, we efficiently introduced mutations in hERG cDNA despite GC‐rich sequences. A pH‐sensitive fluorescent tag was fused to hERG for efficient evaluation of channel trafficking. An optimised 35‐s patch‐clamp protocol was developed to evaluate hERG channel activity in transfected cells. R software was used to speed up analyses.

**Results:**

In the present work, we observed a good correlation between cell surface expression, assessed by the pH‐sensitive tag, and current densities. Also, we showed that the new biophysical protocol allows a significant gain of time in recording ion channel properties and provides extensive information on WT and variant channel biophysical parameters, that can all be recapitulated in a single parameter defined herein as the repolarisation power. The impacts of the variants on channel structure were also reported where structural information was available. These three readouts (trafficking, repolarisation power and structural impact) define three pathogenicity indexes that may help clinical diagnosis.

**Conclusions:**

Fast‐track characterisation of *KCNH2* genetic variants shows its relevance to discriminate mutants that affect hERG channel activity from variants with undetectable effects. It also helped the diagnosis of two new variants. This information is meant to fill a patient database, as a basis for personalised medicine. The next steps will be to further accelerate the process using an automated patch‐clamp system.

## Graphical abstract

We developed a method for rapid analysis of the properties of new hERG channel variants that is helpful for clinicians to diagnose an arrhythmia and help counseling the patient. This method is rapid enough to provide a complete set of channel properties and will set the stage for a more extensive characterization in the future.

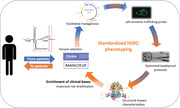

## 1 INTRODUCTION

### 1.1 Cardiac arrhythmias linked to hERG channelopathies

Cardiovascular diseases are the first cause of death, with an estimated 17.9 million losses per year, corresponding to 31% of mortality worldwide (https://www.who.int/health-topics/cardiovascular-diseases#tab=tab_1). Cardiac arrhythmias, often linked to defective ion channels, significantly contribute to the casualties.^1^ Long QT syndrome (LQTS) is a cardiac repolarisation disorder characterised by the prolongation of the QT interval and abnormal T waves in the electrocardiogram.^2^ This inherited arrhythmic disorder has a prevalence of one per 2000 people and is associated with sudden cardiac death.^3^ Three encoding ion channels genes have been identified as responsible for most of the LQTS cases: *KCNQ1* (K_v_7.1 channel) causing LQT1,^4^ *KCNH2* (hERG ‐ K_v_11.1 channel) causing LQT2^5^ and *SCN5A* (Na_v_1.5 channel) causing LQT3.^6^ The hERG channel is distinguished from other voltage‐gated potassium channels by slow activation and fast inactivation.^7^ As a result, the channel is inactivated at the beginning of the action potential (AP), while it opens only gradually when repolarisation starts, resulting in an outward current. This current compensates for the driving force decrease as the membrane potential approaches the potassium ion equilibrium potential. As a result, hERG potassium channels play a key role in cardiac repolarisation. hERG dysfunction (LQT2) is thought to cause a third of all LQTS cases.^2^ Other arrhythmias are linked to a shortening of the ventricular AP^8^ and lead to a short QT syndrome (SQTS1), some of them also implying hERG potassium channels.^9^ SQTS1 variants occur much less frequently than LQT2 variants with about 30 reported cases in Clinvar (https://www.ncbi.nlm.nih.gov/clinvar) and a few confirmed cases in cell electrophysiology.^10^

### 1.2 Pathogenicity of LQT2 variants

Over 1000 mutations in hERG related to LQT2 have now been described in various databases. These include Clinvar at the international level, but also Bamacoeur, a French network (http://www.filiere‐cardiogen.fr/), at the national level. Following clinical diagnosis, tens of new variants are identified yearly. Next generation sequencing demonstrated the extraordinarily high variability of the human genome and in particular the abundance of rare variants (<0.1%) modifying the protein sequence without functional consequences.^11^ In this context, allele frequency can no longer be considered as evidence for variant pathogenicity. In 2015, the American College of Medical Genetics and the Association for Molecular Pathology (ACMG‐AMP) published international guidelines to classify variants in five classes from pathogenic (class 5) to benign variants (class 1).^12^ However, without information related to segregation among relatives or functional studies, variants are often considered of unknown significance (VUS). To date, approximately 19% of known hERG variants have been characterised, all the remaining ones are still VUS. In addition, most of the hERG variants studied so far have been investigated in different laboratories around the world without consensual standardised procedures. This heterogeneity in approaches complicates the classification of hERG variants pathogenicity. Misinterpretation of a VUS can lead to inappropriate genetic counselling within the families and, sometimes, treatment as well.^13^, ^14^

### 1.3 A standardised assessment of the pathogenicity of hERG variants

With the ever‐increasing speed at which genotyping is performed, we now need an efficient methodology to define the pathogenicity of hERG VUS based on functional and structural data. Although several approaches are routinely used for functionally characterising hERG VUS, they are not adapted for large‐scale investigations. Bottlenecks include: (i) difficulties in hERG channel mutagenesis due to GC‐rich regions, (ii) disputable evaluation of channel trafficking by examining glycosylation levels using Western blots (knowing that some mutations can affect glycosylation sites),^14^, ^15^ (iii) slowness of manual patch‐clamp experiments and (iv) difficulties to extract a universal pathogenicity index from complex biophysical analyses. There have been initiatives for large‐scale investigation of hERG variants but these require the production of stable cell lines for each variant^16^, ^17^ or the use of bicistronic plasmids with biased inequivalent expression of the two transgenes in heterozygous conditions.^18^ In addition, none of the large‐scale studies have made use of the reported Cryo‐EM structure of the hERG channel to explore the structural impact of a given variant on hERG. Thus, there is still a need to increase the pace at which hERG VUS are characterised without compromising on the quality of this assessment.

Herein, we propose to explore the pathogenicity of hERG variants on three parameters: (1) trafficking, (2) channel function and (3) local impact of the variant on channel structure. To speed‐up characterisation, we innovated on several technical aspects. First, we implemented a technique for reliable introduction of hERG mutations. Second, we validated the use of a new fluorescent tag for the cell surface trafficking of hERG. Third, we developed a short patch‐clamp protocol that recapitulates all essential information of hERG function and provides a unique index of pathogenicity. Fourth, we explored the quality of a pathogenicity index based on local structural alterations of the channel. To test this approach, we studied 12 LQT and one SQT hERG variants identified in Nantes Hospital patients (France), of which five have never been characterised before. Two of them required urgent phenotyping to help diagnosis. Overall, we propose three hierarchised indexes of pathogenicity that we aim to use as standardised methods to complement the first level of pathogenicity provided by clinical/genetic data to help patients’ risks stratification. We also offer perspectives to transform these new approaches to large‐scale studies by automated patch‐clamp at a later stage.

**HIGHLIGHTS**

- Next‐generation sequencing led to identification of hundreds of new hERG channel variants.
- A combination of robust and fast protocols helps generate and characterise hERG channel variants.
- An optimised 35‐s protocol allows a clinically relevant and a biophysical characterisation of the variants in homozygous and heterozygous conditions.
- A new pHluorin tag helps the study of the trafficking defects of the channel.

## 2 MATERIALS AND METHODS

Classical techniques referring to *hERG variant plasmid generation*, *cell culture*, *plasmid transfection*, *manual patch clamp* and *classical protocols* and *confocal microscopy* are all described in Supplementary Material Online. New protocols and analyses are described herein.

### 2.1 Electrophysiology

For manual patch‐clamp experiments, the optimised protocol is available at dx.doi.org/10.17504/protocols.io.bte3njgn and Supplementary File 2. Calculated junction potential (*V*_m_ = *V*_p_ – 8.2 mV) was considered for AP‐clamp protocol, which was generated using the O'Hara and Rudy model of sub‐epicardial ventricular AP.^19^

### 2.2 Optimised protocol analyses in R

For optimised voltage‐clamp protocol analysis, a R automated routine has been developed to perform rapid and reliable analyses (R script accessible at dx.doi.org/10.17504/protocols.io.bte7njhn and Supplementary File 3). This pipeline allows direct analysis from pClamp acquisition files (Supplementary Material Online, Figure S5). Briefly, each depolarisation step has been isolated using a time conversion rule and stimulation steps of each protocol have been superimposed to fully analyse hERG properties (three human ventricular APs, steady‐state activation, steady‐state inactivation and kinetics of activation, inactivation, recovery from inactivation and deactivation).

### 2.3 Modelling

In 2017, the crystal structure of hERG was resolved (PDB ID#5VA1, 5VA2 and 5VA3). We decided to take advantage of the availability of this structure to visualise in the protein the different locations of the considered mutations.

Using UCSF Chimera, an open access software, we evaluated for each mutation the impact of the surrogate residue on the rest of the protein. We are aware that this approach is approximate since, on the one hand, it is based on a given state of the protein and that, on the other hand, the latter presents absent parts which we do not know if they can be impacted by the different mutations. Nevertheless, by making it possible to categorise these different mutations, it helps, along with electrophysiology, to anticipate the expected impact.

To evaluate this impact for each mutation considered, we propose to address to these mutations a score of 0, 1 or 2 for three different criteria A, B and C where:

Criterion A: evaluates the change in occupied space (In this criterion, a score of 0 = no change, 1 = small change, 2 = large change).

Criterion B: takes in consideration the modification of charge, polarity or water affinity (0 = identical charge/polarity/hydrophilia, 1 = disappearance/appearance of charge/polarity, 2 = opposite charge/polarity/hydrophilia).

Criterion C: evaluates the modifications of interactions with nearby residuals (0 = no clash, 1 = minimisable clash, 2 = non‐minimisable clash).

Since the scores for the first two criteria (A and B) were established independently of the crystal structure of the channel, we relied on substitution tables, the elaboration of which is detailed (Supplementary Material Online). Criterion C is directly linked to the nature of the substituted residues in the context of the channel structure. As such, the score attributed to it can only be measured on a case‐by‐case basis, considering all the amino acids impacted by the mutation under study. The final score, resulting from the addition of each score given for each criterion, should permit to rank each mutation from ‘with no or low impact’ to ‘high or deleterious impact’.

To facilitate the visualisation, we have decided a colour code to categorise and visualise the mutations according to their potential impact: red for a high potential impact (score ≥ 4), yellow for a limited impact (2 ≤ score ≤ 3) but to be considered, and green for a low impact (score < 2).

## 3 RESULTS

## 4 Optimised hERG plasmid for efficient variant introduction

The structure of the hERG channel was published in 2017.^20^ The channel is composed of eight domains from the N‐ towards C‐terminus: the N‐tail domain (amino acids (AA) 1–26), the PAS domain (AA 27–135), a N‐linker domain (AA 136–398) with a major portion missing in the structure (AA 140–341), the VSD (AA 399–545), the pore domain (AA 546–670), the C‐linker domain (AA 671–736), the CNBHD (AA 737–863) and the C‐tail domain (AA 864–1159) from which a significant portion is missing too (AA 870–1006) (Figure S1(A)).

One of the difficulties with hERG mutagenesis is the existence of highly enriched GC regions that hamper the progression and fidelity of DNA polymerase required for DNA amplification. To facilitate mutagenesis, the sequence encoding wild‐type hERG channel (NCBI reference: NM_000238.4) was synthesised along with silent mutation insertions that introduced unique restriction sites for the following enzymes: *Bam* HI, *Kpn* I, *Nsi* I, *BsiW* I and *Not* I in different regions of hERG cDNA (Figure S1(B)). These changes provided opportunities for targeted manipulation of specific regions of this Opti‐hERG ion channel sequence, GC‐rich, namely those located between the inserted *Bam*HI–*Kpn*I and *Bsi*WI–*Not*I restriction sites (∼80% GC content), but also non‐GC‐regions located between other couples of restriction sites. We noticed that mutagenesis of all these regions was dramatically facilitated by performing the polymerase reactions on these isolated regions. Then, the mutated fragments were inserted using the Gibson assembly method.^21^ Using this procedure, mutagenesis was successful in more than 90% of the clones, drastically limiting the need of clone selection. This high success rate is a significant gain of time and limits the costs of cDNA sequencing. With the Opti‐hERG sequence, if by adventure the mutagenesis by PCR is still reluctant in these GC‐rich regions, it is still possible to directly order the synthetic fragment between the two closest silent restriction sites for Gibson assembly.

Finally, to ease the evaluation of the impact of the hERG channel variants on trafficking, a tag sequence was added 3’ of the hERG nucleotide sequence that encodes the transmembrane segment of transferrin followed by the fluorescent pHluorin protein at the C‐terminus of the hERG channel. This tag was successful in evaluating the plasma membrane expression levels of TREK1 and TRAAK channels^22^ compared with the total expression levels. The pH sensitivity of pHluorin (loss of fluorescence at pH 6.0 versus pH 7.4) allows for fluorescence quenching of the channels expressed at the membrane, without affecting fluorescence levels in internal compartments. We named this construct the Opti‐hERG‐pHluorin sequence. The resulting sequence was cloned in the pCDNA5/FRT/TO plasmid between the *Hin*dIII and *Xho*I restriction sites.

Missense mutations were selected from a French hospital patient database (Bamacoeur from the National Cardiogen network including the CHU Nantes Hospital, France). Several criteria of selection were used: (i) a rather large domain coverage, (ii) a mix of previously characterised (eight out of 13; R35W, C64Y, I96T, R176W, R328C, R534C, G548S, A561V) and not characterised (five out of 13; T74R, K93E, D591H, R835P, P1026L) variants, D591H and P1026L being recently identified VUS, of major interest for the cardiologists of our network (Table 1 and Figure S1(B)). Several variants were located in the PAS domain, which has been implicated in both hERG trafficking and functional properties.^15^, ^35^ Twelve of these variants were identified in long QT patients, but our strategy is also tested for a short QT variant (D591H). Hence, a total of 13 hERG variants were constructed for this study.

**TABLE 1:** Variants hERG: location, nucleotide and amino‐acid changes

hERG variant	Nucleotide mutation	Domain	Wild residue	Position	Target residue	Mutation type	Reference
R35W	c.103 C>T	PAS	R	35	W	Missense	^23^, ^24^
C64Y	c.191 G>A	PAS	C	64	Y	Missense	^15^, ^25^, ^26^, ^27^, ^28^
T74R	c.221 C>G	PAS	T	74	R	Missense	^29^
K93E	c.277 A>G	PAS	K	93	E	Missense	None
I96T	c.287 T>C	PAS	I	96	T	Missense	^30^, ^31^
R176W	c.526 C>T	N‐Linker	R	176	W	Missense	^32^, ^33^, ^34^
R328C	c.982 C>T	N‐Linker	R	328	C	Missense	^29^
R534C	c.1600 C>T	VSD	R	534	C	Missense	^29^
A561V	c.1682 C>T	Pore	A	561	V	Missense	^29^
G584S	c.1750 G>A	Pore	G	584	S	Missense	^29^
D591H	c.1771 G>C	Pore	D	591	H	Missense	None
R835P	c.2504G>C	CNBDH	R	835	P	Missense	None
P1026L	c.3077 C>T	C‐tail	P	1026	L	Missense	None

### 4.1 Classical functional analyses of hERG variants by manual patch clamp for use as reference parameters

**FIGURE 1:**
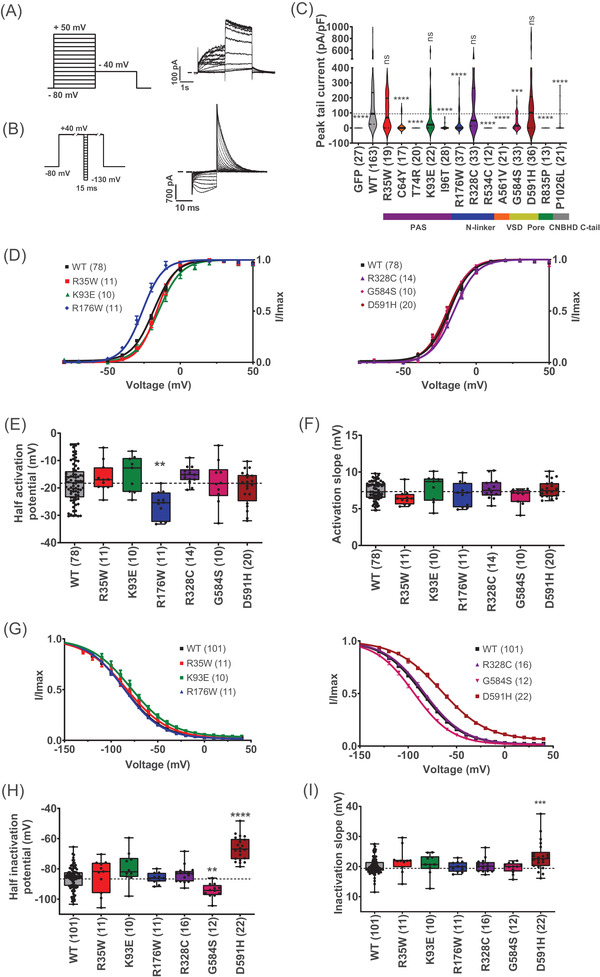
Classical functional analyses of hERG variants by manual patch‐clamp. (A) Left: activation voltage protocol used, one sweep every 8 s. Right: Representative, superimposed recordings of the WT hERG current in CHO cells transfected with hERG (0.8 μg Opti‐hERG‐pHluorin plus 1.2 μg eGFP plasmids). (B) Left: inactivation voltage protocol used, one sweep every 5 s. Right: Representative, superimposed recordings of the WT hERG current. (C) Violin plots of WT and variants maximal current densities extracted from the inactivation protocol. ****p value versus WT hERG <.0001, ***p value versus WT hERG <.001, non‐significance (ns) *p* value versus WT hERG, Kruskal–Wallis test. A dotted line crosses the median of the WT. (D) Activation curves of WT and hERG variants obtained from tail currents using the protocol shown in A, lines are Boltzmann fits to the data. Variants with at least 20% of current amplitude relative to WT were analysed. (E) and (F) Tukey plots of WT and hERG variants half‐activation potential and activation slope, respectively. Black dots represent individual values. (G) Inactivation curves of WT and hERG variants obtained from tail currents using the protocol shown in B, lines are Boltzmann fits to the data. (H) and (I) Tukey plots of WT and variants hERG half‐activation potential and inactivation slope, respectively. ****p value versus WT hERG <.0001, ***p value versus WT hERG <.001 and **p value versus WT hERG <.01, Kruskal–Wallis test

We first established the functional properties of the 13 selected hERG variants using classical protocols in manual patch clamp (Figure 1). The protocols classically used to characterise hERG channel steady‐state activation and inactivation are shown in Figures 1(A) and 1(B), respectively, along with representative current traces for the Opti‐hERG‐pHluorin channel. Of note, the biophysical properties of the Opti‐hERG‐pHluorin were indistinguishable from those of the non‐modified hERG construct expressed by the same plasmid indicating that the neutrality of the pHluorin tag (Table S2 and Figure S2). As another control, and since the study of the variants was performed over a significant period, we tested if the WT Opti‐hERG‐pHluorin current densities were stable during the study. Recording of WT Opti‐hERG‐pHluorin channels were pooled in four different batches, which did not show variations in currents densities when compared one to another (Figure S3). According to the data, variants R35W, K93E, R328C and D591H did not affect the peak tail current density (Figure 1C and Table S3). All other variants (nine out of 13) presented a significant decrease in current density. Of these nine variants, four of them (T74R, R534C, A561V and R835P) had no currents at all, while three others had currents too low for further functional characterisation (C64Y, I96T and P1026L). The remaining two variants (R176W, G584S) presented diminished currents but of sufficient density to be investigated for their functional properties. The decreased current of R176W is consistent with an earlier publication.^36^ Activation curves were built from data generated according to the protocol shown in Figure 1(A). As shown, only variant R176W had activation properties different from the WT Opti‐hERG‐pHluorin channel (Figure 1(D)), with an average *V*_0.5_ of −26.1 ± 1.5 mV (R176W, *n* = 11, *p* ≤ .01) versus *V*_0.5_ of −18.2 ± 0.8 mV (WT, *n* = 78) (Figure 1(E)). No differences were observed for the slopes of activation (Figure 1(F)). Next, inactivation curves were built from the currents generated by the protocol shown in Figure (1B). Two variants presented differences in the inactivation curves compared with the WT (G584S and D591H) (Figure 1(G)). Evaluation of the extracted parameters illustrated a change in *V*_0.5_ of inactivation from −86.2 ± 0.7 mV (WT, *n* = 101) to −94.3 ± 1.3 mV (G584S, *n* = 12, *p* ≤ .01), and −66.4 ± 1.7 mV (D591H, *n* = 22, *p* ≤ .0001) (Figure 1(H)). The *V*_0.5_ shifts towards depolarised values of D591H variant indicated a gain‐of‐function, while the opposite shift of G584S inducing a loss of function. Evaluation of the slope of the inactivation curves show a significant alteration only for the D591H variant (Figure 1(I)). *k* Value was 23.4 ± 1.0 (D591H, *n* = 22, *p* ≤ .001) versus 20.1 ± 0.3 (WT, *n* = 101). The major changes introduced by the D591H are all compatible with the clinical classification for a short QT variant. Noteworthy, the pHluorin tag, as for the WT hERG channel, changed none of the properties of D591H channel (Table S2 and Figure S2).

To enhance the efficiency of trafficking and electrophysiological analyses, we developed new faster protocols, compatible with high‐throughput characterisation of hERG variants.

### 4.2 The pHluorin tag faithfully and rapidly reflects trafficking issues of hERG variants

**FIGURE 2:**
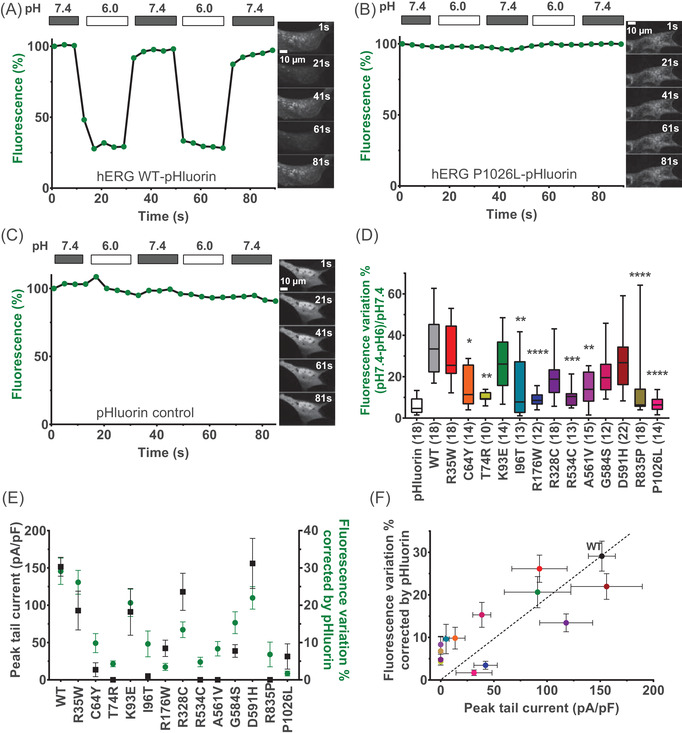
The pHluorin tag faithfully and rapidly reflects trafficking issues of hERG variants (A). Representative recording of pHluorin fluorescence in MDCK cells transfected with WT Opti‐hERG‐pHluorin plasmid, showing fluorescence quenching at pH 6.0 relative to pH 7.4. (B) Representative recording of pHluorin fluorescence in MDCK cells transfected with P1026L Opti‐hERG‐pHluorin plasmid, showing no fluorescence quenching at pH 6.0 relative to pH 7.4. (C) Representative recording of pHluorin fluorescence in MDCK cells transfected with pHluorin control plasmid, showing no fluorescence quenching at pH 6.0 relative to pH 7.4. (D) Tukey plot of mean ± sem percentage of fluorescence variation (pH 7.4–pH 6.0)/pH 7.4, representing the expression of channels in the cell membrane, corrected by basal pHluorin fluorescence. ****p value versus WT hERG <.0001, ***p value versus WT hERG <.001 and **p value versus WT hERG <.01, *p value versus WT hERG <.05, Kruskal–Wallis test. (E) and (F) Comparison of mean ± SEM peak tail hERG current densities from classical protocols (same as in Figure 1(C), black) and mean ± SEM percentage of fluorescence variation (green). In panel (F), coloured points represent the different hERG variants as in panel (D). Dotted line is traced from the origin to the WT point to indicate WT current densities versus fluorescence variation relationship

To evaluate the trafficking of hERG variants at 37°C using a different parameter than current densities, we took advantage of the pHluorin tag. hERG channels were expressed in MDCK cells that have the advantage to be large and flat, facilitating the confocal imaging analyses of channel distribution.^22^, ^37^ In a representative example, we imaged and quantified the fluorescence of WT Opti‐hERG‐pHluorin channel by confocal microscopy (Figure 2(A)). As shown, lowering the extracellular pH value from 7.4 to 6.0 produced a reversible and reproducible ∼70% decrease of the WT Opti‐hERG‐pHluorin channel fluorescence level of the cell imaged. This indicates that there are approximately 2.3‐fold more channels expressed at the plasma membrane than intracellular ones. Performing the same experiment on a cell expressing the P1026L variant, previously classified by us as non‐functional (Figure 1(C)), indicates that the fluorescence level is insensitive to pH change (Figure 2(B)). Hence, this channel type is exclusively expressed intracellularly. The same pH insensitivity was observed in control cells transfected with a pHluorin plasmid, which exhibits only cytoplasmic expression of the fluorescent protein (Figure 2(C)). Analysis of membrane expression of the 13 hERG variants showed a significant reduction of membrane levels for most of them compared with the WT. The absolute values of % fluorescence variation change from 35.1 ± 3.5 (WT, *n* = 18) to 15.5 ± 2.6 (C64Y, *n* = 14, *p* < .05), 10.3 ± 0.8 (T74R, *n* = 10, *p* < .01), 14.3 ± 3.8 (I96T, *n* = 13, *p* < .001), 9.2 ± 1.0 (R176W, *n* = 12, *p* < .0001), 10.6 ± 1.3 (R534C, *n* = 13, *p* < .001), 14.3 ± 2.0 (A561V, *n* = 15, *p* < .01), 12.4 ± 3.7 (R835P, *n* = 18, *p* < .0001), 6.6 ± 0.9 (P1026L, *n* = 14, *p* < .0001). Five variants (R35W, K93E, R328C, G584S and D591H) remain with membrane expression levels like WT (Figure 2(D)). Next, to confirm that the fluorescence change is a good indicator of membrane level, we compared the fluorescence variation induced by pH change with the current densities measured for the hERG variants (Figure 2(E)). The variations observed in both parameters were nicely consistent indicating that the pHluorin tag is valuable for efficient evaluation of plasma membrane expression of the hERG channel. This was confirmed by investigating how both paradigms correlate (Figures 2(E) and 2(F)). Channel variants with no currents had either no fluorescence change (6% background fluctuation) or presented a slight fluorescence change indicating that a small fraction of these channels reached the plasma membrane but were non‐functional (A561V for instance, which is logical because located in the pore region). Of course, preserved membrane expression does not prejudge the functionality of the correctly addressed channel variants and patch‐clamp experiments are needed to conclude on that matter.

### 4.3 A new optimised voltage‐clamp protocol for complete and fast‐track extraction of hERG channel parameters

Pathogenicity of hERG variants can be explained by multiple forms of alterations of the channel biophysics (steady‐state activation or inactivation, kinetics of activation, inactivation, deactivation and recovery from inactivation, current amplitude, ionic selectivity). For any of these parameters, if a variant introduces a form of pathogenicity, it should be readily visualised by the K^+^ exit from the channel during an AP. Moreover, acquiring hERG channel parameters using classical voltage‐clamp protocols is lengthy to acquire (more than 10 min) and cumbersome to analyse. We strived for improving the acquisition of hERG channel parameters using an optimised voltage‐clamp protocol (Figure S4). This new optimised protocol lasts 35 s and encompasses seven subprotocols. The first subprotocol (SP1) is composed of 3 human ventricular APs generated *in silico* (from −88 to 36 mV overshoot with a 1 s cycle length). These APs are repeated to make sure to reach steady state regarding hERG gates (no run‐down or run‐up). This equilibrium is achieved at the second and third APs. It is followed by six especially designed subprotocols to reduce the acquisition time at its maximum. For steady‐state activation (SP2), we used various depolarisation times adapted to the hERG kinetics of activation at each potential (at least for the WT): longer depolarisation times when activation develops slowly (e.g., −50 mV) and shorter ones when activation develops quickly (e.g., +70 mV). These durations were based on the time constants of activation measured at various potentials. An additional novelty is that inactivation is relieved fully before hERG current measurement at +70 mV. This allows measuring currents of higher amplitudes (about 10‐fold higher) than in classical protocols, which can be helpful for mutations with decreased current amplitude. For steady‐state inactivation (SP3), we depolarised to +70 mV only once to fully open the channels, and we used varying potentials (−130 to +70 mV) to measure the inactivation level with pulses brief enough that keep the activation level constant. When deactivation was observed (generally for the two last pulses), a calculation was performed to compensate for this level of deactivation. The next four subprotocols are meant to measure kinetics of recovery of activation (SP4), inactivation (SP5), recovery of inactivation (SP6) and activation (SP7). The last subprotocol allowed for measuring activation at 70 mV, first, then at 0 mV, second. Although this global protocol was designed for fast‐track characterisation of hERG variants, it may also prove useful in the future for determining the mechanism of action of a pharmacological drug.

### 4.4 The repolarisation power as unique index of pathogenicity

**FIGURE 3:**
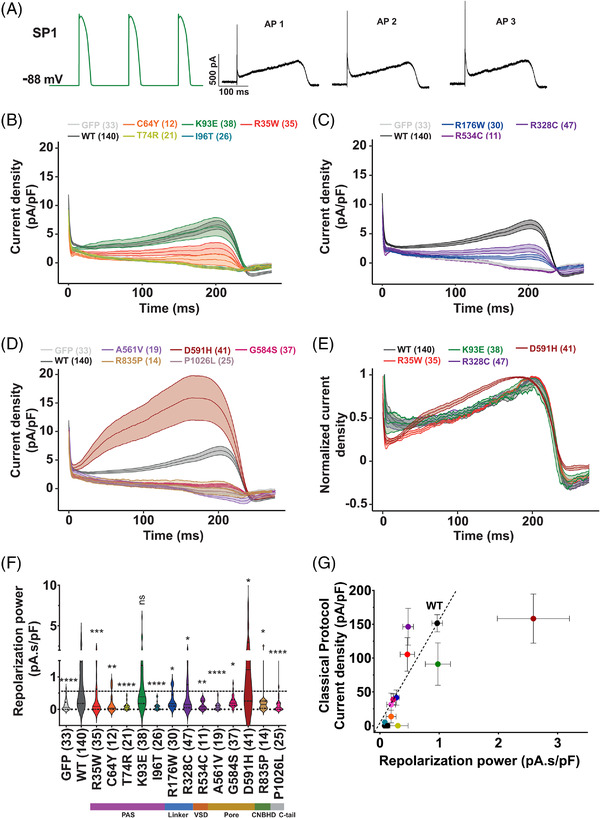
Action potential clamp (AP‐clamp) and repolarisation power. (A) Left: the ventricular action potential voltage subprotocol used (Figure S4 SP1). The small inward current observed when the action potential is returning to resting values is attributed to a contamination of the intracellular solution by the extracellular Tyrode solution. This occurred despite liquid junction potential and leak current subtraction. Right: Representative recordings of the WT hERG current in CHO cells transfected with hERG (0.8 μg Opti‐hERG‐pHluorin plus 1.2 μg eGFP plasmids) during stimulation shown in left. Three successive AP‐clamp protocols were recorded to test for current stability, the last one was used for analysis. (B–D) Mean ± SEM current density recordings during action potential clamp. (E) Normalised current density recordings for variants showing current densities of at least 40% of WT. (F) Violin plots of the time integral of the recorded currents densities, named repolarisation power, of WT and hERG variants. ****p value versus WT hERG <.0001, ***p value versus WT hERG <.001, **p value versus WT hERG <.01, *p value versus WT hERG <.05, non‐significance (ns) *p* value versus WT hERG, Kruskal–Wallis test. A dotted line crosses the median of the WT. (G) Relationship of mean ± SEM peak tail hERG current densities from classical protocols (same as in Figure 1(C)) and mean ± SEM repolarisation power. Same colour code of hERG variants as in panel (F)

As mentioned above, the repolarisation power is an interesting index to summarise all kinds of biophysical changes introduced by a given hERG channel variant. Representative currents for the WT hERG channel in response to all three APs used in the first subprotocol (SP1) are shown in Figure 3(A). Steady state was reached since variation in current amplitude between AP 2 and 3 was systematically less than 10% (*n* = 927 cell recordings) indicating that three APs were enough to reach stability. We next compared the hERG currents during the third AP clamp between WT hERG and variants in the PAS domain (Figure 3(B)), the N‐linker and VSD (Figure 3(C)) and the pore, CNBDH and C‐linker (Figure 3(D)). For most variants, the average current level during AP was lower than WT. Two exceptions were observed: K93E behaved similarly to WT, whereas D591H produced more current. Several variants did not produce any current (compared to eGFP‐positive cells alone): C64Y, T74R, R534C, A561V, R835P and P1026L. We normalised all these traces to the maximal peak current to examine a possible change in their kinetic profiles (Figure 3(E)). Altered profiles may impact the shape of the AP regardless of the current amplitude. Remarkably, the D591H variant produces a profound alteration in profile of the current that peaks at 0.18 s, compared with 0.20 s for WT and other variants. Using these currents, we defined a repolarisation power of the WT and hERG variants by integrating them as a function of time (Figure 3(F)). According to the representation, we observed that the K93E variant has a similar repolarisation power as WT hERG and that the D591H has a much higher repolarisation power. Other variants have lower repolarisation powers, including R35W and R328C, which were not showing a significant decrease in maximal tail current densities extracted from the inactivation protocols (Figure 1(C)). The fact that these two variants show a decreased repolarisation power is probably due to the higher sample size (35 and 47 versus 19 and 33, respectively). For D591H, the fact that the repolarisation power includes the shift in the inactivation curve, explains why it is significantly increased as compared to WT, whereas maximal tail current densities extracted from the inactivation protocols were not different (Figure 1(C)). We correlated the current densities measured in Figure 1(C) with the repolarisation power of Figure 3(F) (Figure 3(G)). This further illustrates that D591H is excluded from the correlation, as this variant exhibits a major gain‐of‐function due to a shift in the inactivation curve. As such, it should not be directly correlated with the current density measured in a classical protocol.

### 4.5 An optimised activation protocol maximising steady‐state equilibrium

**FIGURE 4:**
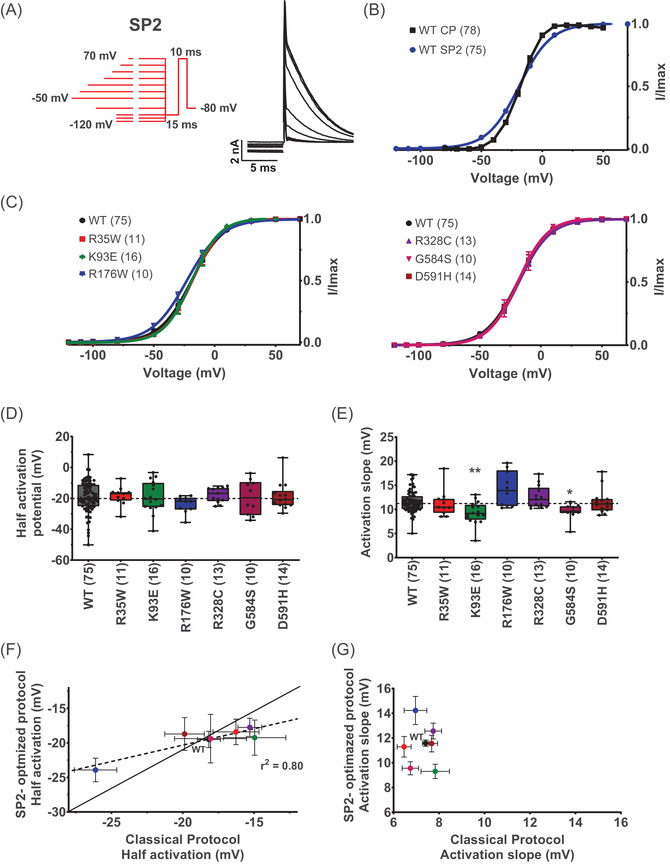
Steady‐state activation subprotocol maximising steady‐state equilibrium. (A) Left: Steady‐state activation voltage subprotocol used (Figure S4 SP2). Right: Representative, superimposed recordings of the WT hERG current in CHO cells transfected with hERG (0.8 μg Opti‐hERG‐pHluorin plus 1.2 μg eGFP plasmids). (B) Comparison of activation curves of WT hERG obtained from tail currents using the protocol shown in panel A (WT SP2) and the classical protocol shown in Figure 1(A) (WT CP). Lines are Boltzmann fits to the data. (C) Activation curves of WT and variant hERG obtained from tail currents using the protocol shown in (A), lines are Boltzmann fits to the data. (D) and (E) Tukey plots of WT and hERG variants half‐activation potential and activation slope, respectively. Black dots represent individual values, and a dotted line crosses the median of the WT. Variants with at least 20% of current amplitude relative to WT were analysed. **p value versus WT hERG <.01, *p value versus WT hERG <.05, Kruskal–Wallis test. (F) and (G) Correlation between SP2 optimised protocol versus classical plots of WT and hERG variants half‐activation potential and activation slope, respectively. Coloured points represent different variants hERG as panels (D) and (E). Dotted line represents the linear regression (*r* ^2^ = 0.80) and the solid line (*y* = *x*) represents equality between half‐activation values obtained from the two protocols

Since activation kinetics depends on membrane potentials, we used depolarisation steps of optimised durations, longer for low voltages and shorter for high voltages (Figure 4(A), see protocol and representative traces). Using such a subprotocol (SP2), we visualise properties much closer to steady state with shallower activation curve, compared with the classical protocol used (Figure 4(B)). Mean *V*_0.5_ values were −19.5 ± 1.2 mV (SP2, WT Opti‐hERG‐pHluorin, *n* = 75) versus −18.2 ± 0.8 mV (classical protocol, WT Opti‐hERG‐pHluorin, *n* = 78), and *k* were 11.6 ± 0.3 mV (SP2) versus 7.4 ± 0.1 mV (classical protocol). This subprotocol 2 has a more realistic assessment of the steady‐state activation properties of the hERG channel. Using this subprotocol, we compared the activation curves of the hERG variants (Figure 4(C)). With this protocol, R176W does not show half‐activation potential different from the WT Opti‐hERG‐pHluorin channel (Figures 4(D) and 4(F)), indicating that the 2‐s long activation steps used for the classical activation protocol are not long enough to reach steady‐state values. An earlier study, using 4‐s steps instead of 2 s illustrates that indeed the R176W variant is not different from wild‐type in activation properties.^32^ This result indicates that the mini‐protocol is adapted to evaluate voltage‐dependence of activation at steady state. Two variants, K93E and G584S, have slightly but significantly lower slope values of activation (Figure 4(E)). As expected from a protocol that extracts optimised parameters for activation, *k* values were systematically higher with the optimised protocol, justifying its use also for the variants (Figure 4(G)).

### 4.6 A fast inactivation protocol with a single activation step

**FIGURE 5:**
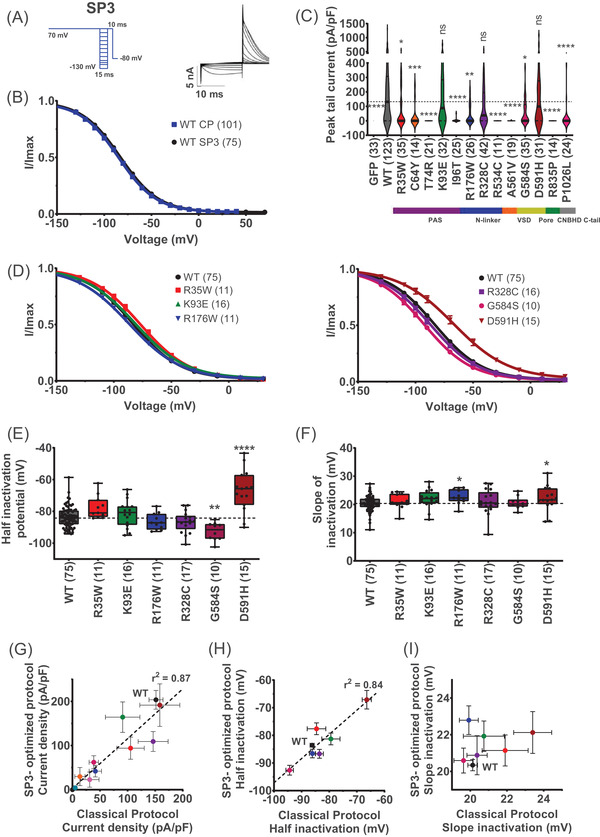
Steady‐state inactivation subprotocol. (A) Left: Steady‐state inactivation voltage subprotocol used (Figure S4 SP3). Right: Representative, superimposed recordings of the WT hERG current in CHO cells transfected with hERG (0.8 μg Opti‐hERG‐pHluorin plus 1.2 μg eGFP plasmids). (B) Comparison of inactivation curves of WT hERG obtained from tail currents using the protocol shown in A (WT SP3) and the classical protocol shown in Figure 1(B) (WT CP), lines are Boltzmann fits to the data. (C) Violin plots of WT and variants hERG maximal current densities extracted from the inactivation protocol SP3. A dotted line crosses the median of the WT. ****p value versus WT hERG <.0001, ***p value versus WT hERG <.001, and **p value versus WT hERG <.01, *p value versus WT hERG <.05, non‐significance (ns) *p* value versus WT hERG >.05, Kruskal–Wallis test. (D) Inactivation curves of WT and variants hERG obtained from tail currents using the protocol shown in A, lines are Boltzmann fits to the data. (E) and (F) Tukey plots of WT and hERG variants half‐inactivation potential and inactivation slope, respectively. Variants with at least 20% of current amplitude relative to WT were analysed. Black dots represent individual values, a dotted line crosses the median of the WT. ****p value versus WT hERG <.0001, **p value versus WT hERG <.01, *p value versus WT hERG <.05, Kruskal–Wallis test. (G), (H) and (I) Correlation between SP3 optimised protocol versus classical plots of WT and variants hERG current density, half‐inactivation potential and inactivation slope, respectively. Coloured points represent different variants hERG as panels (E) and (F). Simple linear regression *R* squared *r* ^2^ = 0.87 (G), and *r* ^2^ = 0.84 (H)

In our optimised subprotocol N°3 (SP3), the inactivation is studied with a single activation step to fully open the channels before evaluating inactivation per se (Figure 5(A), see subprotocol and representative traces). Noteworthy, with this faster inactivation subprotocol, we keep the quality of assessment of hERG channel inactivation properties of the classical protocol (Figure 5(B)). Mean *V*_0.5_ values were −83.6 ± 0.7 mV (SP3, WT Opti‐hERG‐pHluorin, *n* = 75) versus −86.2 ± 0.7 mV (classical protocol, WT Opti‐hERG‐pHluorin, *n* = 101), were significantly different by *p* < .05 (Mann–Whitney test) but were still very similar with less than 3 mV difference. *k* Values were 17.8 ± 0.3 mV (SP3) versus 20.3 ± 0.3 mV (classical protocol), but not significantly different. As for the classical protocol, current amplitudes for WT and all variants were extracted from the maximal value of the Boltzmann fit of the inactivation protocol. Variants K93E, R328C and D591H did not affect the peak tail current density (Figure 5(C)). All other variants (10 out of 13) showed significantly reduced current densities. Of these nine variants, four of them (T74R, R534C, A561V and R835P) still had no currents at all, while three others again had currents too low for functional characterisation (C64Y, I96T and P1026L). The remaining three variants (R35W, R176W and G584S) presented diminished currents but of sufficient density to be evaluated for their functional properties. Inactivation curves were built (Figure 5(D)). In agreement with the earlier assessments, two variants G584S and D591H presented different inactivation curves compared with the WT. A change half inactivation potential values were observed: −92.6 ± 1.8 mV (G584S, *n* = 10, *p* ≤ .01), and −67.1 ± 3.3 mV (D591H, *n* = 20, *p* ≤ .0001) versus −83.6 ± 0.7 mV (WT, *n* = 75) (Figure 5(E)). D591H variant, but also R176W presented a minor but significant change in *k* value with *k* = 22.1 ± 1.1 (D591H, *n* = 15, *p* ≤ .05), *k* = 22.8 ± 1.1 (R176W, *n* = 11, *p* ≤ .05) versus 20.3 ± 0.3 (WT, *n* = 75) (Figure 5(F)). As foreseen from the similar inactivation curve of WT Opti‐hERG‐pHluorin, extracted from both protocols, we observed a good correlation between the extracted parameters, current densities (Figure 5(G)) and *V*_0.5_ (Figure 5(H)).

### 4.7 Global kinetic parameters extracted from the optimised protocol

**FIGURE 6:**
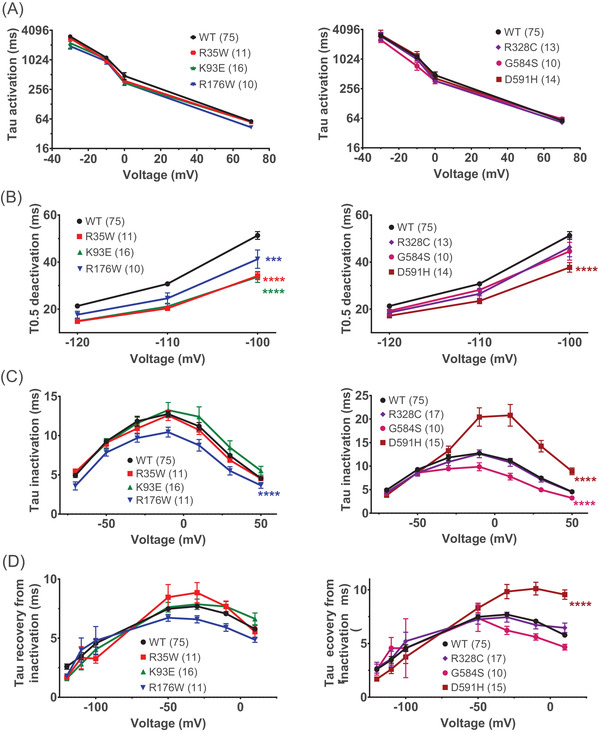
Kinetic parameters extracted from the optimised protocol. Kinetics subprotocols used (SP4–SP7) and representative superimposed recordings are present in Figure S4 and S5 in Supplementary File 1. Variants with at least 20% of current amplitude relative to WT were analysed. (A) Mean ± SEM of activation tau for several membrane potentials. (B) Mean ± SEM of deactivation *T*_0.5_ for several membrane potentials. ****p value versus WT hERG <.0001, ***p value versus WT hERG <.001; two‐way ANOVA test. (C) Mean ± SEM of inactivation tau for several membrane potentials. ****p value versus WT hERG <.0001; two‐way ANOVA test. (D) Mean ± SEM of recovery from inactivation tau for several membrane potentials. ****p value versus WT hERG <.0001; two‐way ANOVA test

As stated above, the fast‐track protocol has also been optimised to encompass most hERG channel parameters, including kinetic ones. Extracting such kinetic parameters in more classical standard ways would require a minimum of four additional protocols which would be incompatible for large scale variant characterisation in stable recording conditions. Activation time constants as a function of voltage were extracted from the subprotocols N°7 (SP7) (0 and 70 mV) and N°2 (SP2) (−30 and −10 mV) (Figure S4 and Figure S5 SP7). Time constants for activation did not differ between variants and WT Opti‐hERG‐pHluorin for all variants presenting sufficient current amplitudes for robust trace fitting (Figure 6(A)). Deactivation time constants at −120, −110 and −100 mV were extracted from subprotocol N°4 (SP4), as illustrated (Figure S5 SP4). Four variants had statistically significant faster deactivation kinetics (R35W, K93E, R176W and D591H), which is consistent with a loss of function on hERG current (Figure 6(B)). Next, we assessed the inactivation kinetics of the channel variants according to the subprotocol N°5 (SP5), as illustrated by the representative traces (Figure S5 SP5). Three variants behaved differently from the WT Opti‐hERG‐pHluorin channel (Figure 6(C)). Two of them have a faster inactivation (R176W and G584S), consistent with a loss of function of hERG current, while D591H had a considerably slower inactivation which leads to a gain‐of‐function. Finally, the time constants of recovery from inactivation were obtained by subprotocol N°6 (SP6) as shown (Figure S5 SP6). Only the D591H variant differed significantly in its recovery from inactivation by being slower than the WT channel. These effects were mostly obvious at more depolarised potentials (above −50 mV) (Figure 6(D)).

### 4.8 Evaluation of the dominant negative effect of four new variants using both the optimised patch‐clamp protocol and pHluorin imaging in heterozygous conditions

The American College of Medical Genetics and Genomics^12^ recommends to use heterozygous conditions for investigating the negative dominant effects of hERG variants. Out of the five new variants that we characterised, we selected three variants that had no currents and that potentially have dominant negative effects (T74R, R835P and P1026L). We added the I96T variant that was already studied by patch clamp, essentially in *Xenopus laevis* oocytes, but without any quantification of the current amplitude and no study of the heterozygous condition.^38^ The functional pathogenicity index, as well as the biophysical properties, of these four variants in heterozygous conditions were studied using the optimised protocol. Figures S6(A) and S6(B) show that co‐expression of three of variants (T74R, I96T and R835P) with WT hERG lead to dominant negative effects. On all other parameters investigated, it was concluded that these four variants had no significant effects on voltage‐dependence of activation and inactivation (Figures S6(C)–S6(H)) except a small, but significant change in the slope of activation for the R835P variant (Figure S6(F)). In addition, none of the kinetic parameters of activation, deactivation, inactivation and recovery from inactivation were modified (Figure S7). The only exception to this was the T74R variant for which inactivation was slightly accelerated.

We also investigated the dominant negative effects of the same four variants using the pHluorin trafficking modality. For these experiments, hERG channel variants, without pHluorin tag, were co‐expressed with WT Opti‐hERG‐pHluorin channels in MDCK cells. Two variants (T74R and R835P) acted as dominant‐negative for the trafficking of the WT channel and these results are consistent with the functional data (Figure S8(A)). The I96T variant does not affect WT trafficking, while it affected the peak tail current density suggesting that this variant impairs only function (Figure S8(B)). Finally, the P1026L non‐significantly affected both the function and trafficking of WT channel. By checking how function and trafficking correlate in heterozygous conditions, there is a clear mismatch for I96T, as described above (Figure S8(C)).

### 4.9 Modelling tool for evaluating the impact of a variant on hERG channel structure

The crystal structure of hERG was resolved in 2017.^20^ It therefore represents an opportunity to evaluate for each variant the impact of the surrogate residue on the rest of the protein. To this end, we used the open access UCSF Chimera software to assign a score of structural impact based on three criteria (first, space occupied by the new amino acid; second, the modification of charge, polarity or water affinity; and third, modifications of interactions with nearby residues). For each of these criteria, a score of severity was assigned from 0 (no change) to 2 (maximal change) (see description in *Methods* section and Supplementary Material Online). The final score is the sum of the three criteria scores. It should allow ranking of each variant from ‘no impact’ (score of 0 to 1) to ‘high impact’ (score of at least 4). The structural impacts of variants were investigated. Three variants belong to structural regions that were not resolved in the Cryo‐EM structure (R176W (N‐linker domain), R328C (N‐linker domain), P1026L (C‐tail domain)) and therefore could not be studied. It should be understood that this approach has an obvious meaning for those variants that will display a high structural pathogenicity index. Those variants with low structural pathogenicity indexes will be of limited information value because this analysis has its limits (no information on subunits, associated proteins, on channel regulation).

We illustrate the example of the structural impact of one variant with high severity.

**FIGURE 7:**
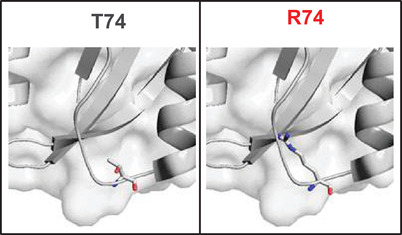
In silico evaluation of the structural impacts of one hERG variant of high pathogenicity. T74R occurs in the PAS domain and received a score of 4, indicating a high impact on hERG structure

The T74R variant occurs in the PAS domain (Figure 7). The substitution of threonine 74 by an arginine residue will, on the one hand, introduce a moderate change in steric occupancy of Δ*V* = 94 Å^3^ (hence a notation of 1 on the first criterion) and, on the other hand, introduce positive charges in the vicinity of a histidine (notation 1 on the second criterion).

The Thr74 residue projects into a pocket located between the Gβ sheet and the Fα sheet of the PAS domain. A manuscript of 2013^39^ showed that this cavity is important for the binding of regulatory molecules (flavins for Phot‐LOV1 phototropin) and that the number of polar residues can be determinant for its ability to detect and bind signals that will modulate its activity. In this pocket, and in the absence of any other molecule, the space is shared between the side chains of Thr74, His70 and Phe98 residues. Still according to the manuscript cited above, His70 seems to be a determining factor in the sensor role of this domain. Among the 34 rotamers proposed by the Chimera application, none can accommodate all the clashes listed. The number of clashes before minimisation ranges from 38 to a minimum of 11 contacts, a notation of 2 is attributed to the third criterion. This mutation therefore suggests a significant impact on the activity or regulation of the channel. A total score of 4 is assigned to this variant.

The full description of all other variants is provided in the Supplementary File 1.

### 4.10 Scoring of trafficking, repolarisation power and structural impacts characterises the pathogenicity of hERG variants

We synthesised all the impact scores for three main hERG properties (modelled structural impact, function as assessed by repolarisation power and channel trafficking) (Table 2). To keep a notation that follows the ACMG recommendations, we arbitrarily arranged the notations for function and trafficking from 1 to 5 as function of severity (0–20% change: 1; 20–40%: 2; 40–60%: 3; 60–80%: 4; and 80–100%: 5) and generated structural maps with colour coding of the pathogenic variants for each parameter assessed (Figure S9). Table 2 provides several interesting information and comparison with previous ACMG classification and Schwartz score, as well as clinical data. First, the modelling data should be performed on solidly resolved hERG structure and not on modelled parts, as in the case of the R35W variant. The R35W mutation predicted an important structural alteration with a score of 4 that is not correlated with high scores for function and trafficking. In contrast, the T74R mutation was structurally assessed on more solid structural data. It is of interest that the score of severity of 5 is very well correlated with the scores observed for function (5) and trafficking (4). It can be predicted therefore that this variant is highly pathogenic. This was confirmed in heterozygous conditions. Concerning the G584S mutation, we obtained a structural score of 3 that was well correlated with a score of 4 for repolarisation power. Regarding trafficking, there is lower score of 2, not well correlated with the repolarisation power, which perfectly illustrates that a variant may impact trafficking to a lower extent than function. This indicates that the trafficking scores complements the repolarisation score for a thorough mechanistic explanation of variant pathogenicity. Finally, the I96T is informative for another reason. The structural score of 1 would predict a low impact on function and/or trafficking. Yet, both function and trafficking were affected (5 for function and 3 for trafficking), also confirmed in heterozygous conditions for function. This indicates that this mutation, while not necessarily impacting hERG channel structure, may alter function through another mechanism. One possibility is that this mutation affects the interaction of the channel with a cellular component whose structural impact is not considered by our current structural assessment. Hence, caution should be brought to the interpretation of structural impact scoring. High scoring, on solid and well‐resolved channel structure, appears as reliable predictors of channel functional alterations. However, low scoring may miss important information regarding channel interactions with other cell constituents. There is an interesting exception to the prediction capability of high structural scoring. In the case of K93E, a high structural score was predicted (4) that was not at all corelated to similar functional or trafficking scores (both 1). This indicates that there are structural regions of the hERG channel that are not important for function (thus missed by the two parameters set here) and that may be important for other non‐assessed functions. These types of variants may nevertheless represent true pathogenic variants but probably require a more complete set of characterisation in native environments (such as the use of cardiomyocytes derived from induced pluripotent stem cells).

**TABLE 2:** The impact scores for three main hERG properties: modelled structural impact, repolarisation power and channel trafficking

Mutation	Modelling score	Repolarisation power score	Trafficking score	ACMG class	Schwartz score	Age (years)	Sex	QTc (ms)	Symptoms	Trigger	T wave	ECG	Test	Family member(s) with definitive LQTS	LQT diag.	Other diagnosis
R35W	4	3	1	5	0	41	M	396	None				Negative mental stress test	No	No	
C64Y	4	5	3	5	4	10	M	509	Fainting		LQT2			Yes	Yes	
**T74R**	4	5 (4)	4 (3)	5	nd											
**K93E**	3	1	1	3	3	49	M	459	Sudden death	Stress				No	No	Hypokinetic cardiomyopathy/Myocardial fibrosis
**I96T**	1	5 (2)	3 (1)	5	5	17	F	492	Syncope	Rest				Yes	Yes	
					4.5	15	M	564	None		LQT2	Bradycardia	Negative exercise stress testing	Yes	Yes	
R176W	Not visible	4	4	5	7	28	F	570	Syncope	Exercise		*Torsade de pointes*		No	Yes	
					1.5	54	M	420	None		LQT2	Bradycardia		Yes	Yes	
					1	12	M	407	Fainting	Exercise	LQT2		Positive mental stress test	Yes	Yes	
					1	14	F	386	None		LQT2			Yes	No	
					3	36	F	495	None				Epinephrine stress test		Yes	
R328C	Not visible	3	2	2	3	29	F	485	None		LQT2			No	Yes	
					2	10	M	436	Syncope	Exercise				No	No	
					0	63	F	448	Fainting	Rest				No	No	
R534C	4	5	4	5	1	37	M	450	None			Bradycardia		No	No	
					2	39	M	450	None		LQT2		Positive mental stress test	Yes	Yes	
A561V	2	5	3	5	4	30	F	575	None					Yes	Yes	
					4	8	M	470	Syncope	Rest		Bradycardia		Yes (sudden death <30 years)	Yes	
G584S	3	4	2	5	4.5	12	M	470	Syncope	Rest	LQT2	Bradycardia	Negative exercise stress testing	Yes	Yes	
D591H*	2	2	1	3	NA	15	M	421	Syncope	Rest				No	No	Short QT
**R835P**	4	4 (3)	4 (3)	5	1	44	F	440	Sudden death	Hypokaliemia			Negative Flecainide test	No	Yes	
**P1026L**	Not visible	5 (1)	5 (2)	3	4	58	M	531	Chest pain				Negative exercise stress testing	Yes	Yes	

Clinical data are also shown. Variants that are characterised for the first time but also I96T (cf text) are in bold. Those that are underlined are variants that were also studied in heterozygous conditions (see Figures S6–S8) and the corresponding scores given in parentheses for the heterozygous conditions. For the functional scores, low pathogenicity is in green, intermediate one in yellow and severe in red. *, of note, D591H was identified as a short QT in the course of this study and the functional scoring would require further refinement to include short QT syndrome variants.

## 5 DISCUSSION

This project was initiated with two main goals in mind: (i) define a protocol that speeds up the phenotyping of new hERG variants to facilitate clinical diagnosis, and (ii) study as many channel properties as possible, compatible with rapid analysis.

### 5.1 Speeding up hERG variant phenotyping

Concerning this first aim, several developments were performed. First, the introduction of new variants within the cDNA encoding the hERG channel is hampered by the high GC‐rich content of the sequence in at least two defined regions (Figure S1(B)). We faced major difficulties in mutating hERG cDNA plasmid by classical mutagenesis approaches using DNA polymerase replication of the full‐length sequence. This is due to the formation of secondary structures like hairpins and higher melting temperatures. To circumvent these difficulties, we introduced five silent restriction sites that allows us to work on parts of the cDNA sequence, including those that are GC rich, and we used the Gibson assembly process to efficiently assemble the three generated fragments: the major fragment of the plasmid, along with the two fragments containing the mutation. The efficacy of this method is excellent (100% success rate on 70 variants generated so far) and considerably speeds up the generation of a database of hERG variants. Of note, one approach that consists in codon optimisation, to reduce the G/C content without altering the amino‐acid sequence, was not chosen because of earlier evidence that it affects channel expression levels. Indeed, an average G/C content reduction from 66 to 51% leads to marked decrease in translation efficiency and a ∼3‐fold decrease in protein expression.^40^, ^41^ Second, we introduced a pHluorin tag to the C‐terminus of the hERG channel to evaluate the percentage of channels that reach the plasma membrane. The approach was validated for the first time on this channel type and shown to reliably explain most of the data from current density measurements. It also appears as an interesting tag for evaluating dominant‐negative effects for the trafficking of WT hERG channels and distinguishing a dominant‐negative effect on traffic from a pure dominant‐negative effect on gating as exemplified by the I96T variant, fully described herein. Changing the pH value in the extracellular medium only marginally affects the intracellular fluorescence from channels that did not reach the plasma membrane, indicating that the approach might be adapted to plate readers for measuring average values of hERG channel membrane expression. Importantly, the pHluorin tag did not modify the biophysical parameters of the WT and a variant hERG channel. The pHluorin approach for assessing membrane expression levels of the hERG channel is comparatively faster than other systems in use nowadays: (i) the evaluation of the relative glycosylation level of the channel by Western blotting^15^ and (ii) the introduction of a α‐bungarotoxin binding site within an extracellular loop of the hERG channel for quantification of fluorescent toxin binding.^42^ Third, we managed to build a biophysical protocol that allows the extraction of ten hERG channel parameters within 35 s of channel recordings. This protocol allows a full characterisation of each cell without taking the risk of an alteration of recording quality during the acquisition (not rare during a 10‐min recording period). Such a fast protocol allows also more recordings per day and more robust statistical analyses. In addition, we managed to develop an automated analysis program under R that is freely available upon request or accessible (Supplementary Material Online).

Other efforts to develop short protocols have been published recently.^43^, ^44^ These authors use either sinusoidal or staircase voltage protocols to characterise hERG channels kinetics, the latter being interesting because it can be adapted for use in high‐throughput automated patch‐clamp systems. With both protocols, the recorded currents were fitted by a Hodgkin Huxley model able to satisfyingly predict the data generated by eight different classical protocols. This elegant mixed experimental/modelling approach was validated for the wild‐type hERG channel so how well it is adaptable to hERG variants is unknown. In addition, in the Lei et al.^43^ study, this short protocol approach was challenged against an activation protocol with steps of 1 s but no a longer one that would have allowed to reach steady state. While these initiatives are all of interest, they are based at least partially on modelling approaches that inherently possess a level of uncertainty. Our optimised protocol avoids this potential bias by exclusively relying on experimental data. In addition, we illustrate that the optimised protocol generates reliable data to describe hERG channel properties because it allows reaching steady state of the activation gate at any potential.

### 5.2 Exhaustive and standardised characterisation of hERG properties

Concerning this second aim, part of our ambition was filled by the evaluation of both cell trafficking and the complete assessment of channel biophysical properties. A major purpose was to define a simple paradigm that recapitulates the entire set of biophysical modifications and the trafficking issues induced by channel variants. We found that applying to the cells an AP that best mimics a physiological ventricular AP would give direct information to the amount of K^+^ ions that exit the cells through the channel variant. This paradigm is the most physiological and integrates naturally all the possible functional alterations produced by the variant, including channel density, open channel probability and various voltage‐dependent and kinetics parameters. Of course, it also integrates the membrane trafficking issues. Interestingly, it also efficiently pinpoints variants that produce gain‐of‐function, with a remarkable 2.6‐fold increase in repolarisation power of D591H variant. This leads us to think that the repolarisation power may become a convenient assessment paradigm for characterising hERG variants. One of the current limits of the repolarisation power is that it is based on a standard AP of a cardiomyocyte that has a normal hERG channel phenotype. In physiological conditions, the AP duration directly depends on the heart rhythm. For the sake of simplicity, we used a unique AP shape, simulated at cycle length of 1 s using the O'Hara model.^19^ Some variants may escape the evaluation of pathogenicity because their impact may be revealed at other cycle lengths. Hence, in the future, it may be of interest to challenge hERG variants with AP of different physiological durations to best uncover the arrhythmia risks. Another limitation of our study is of course the fact that the repolarisation power is determined at room temperature and hERG channel properties are greatly impacted by temperature.^45^ To be fully meaningful, the repolarisation power will need to be evaluated at 37°C despite a lower gigaseal frequency (15% instead of 80%).^46^ This challenge may be more easily addressed using automated patch‐clamp techniques that allow for a much larger set of recordings per unit of time.

Since the publication of hERG channel structure, an additional parameter can be included in the evaluation of variant pathogenicity. We implemented a simple method for the evaluation of the structural impact of a variant on channel structure. This approach is valid for residues that were resolved in the CryoEM structure. Some structural parts of hERG channel are still missing and hence if the variant belongs to these regions, then the method is not applicable. In addition, this approach remains incomplete because it does not assess the impact of a variant onto the interaction of the hERG channel with protein partners, nor the impact of channel regulation. Also, some variants, that belong to structurally defined hERG regions, may hypothetically interact with hERG domains that have not been structurally characterised. Therefore, this approach provides partial but valuable information on pathogenicity and, as mentioned above, we anticipate that the structural index is more robust when it is high and hence suggests pathogenicity. While this new index of pathogenicity is innovative in the characterisation of hERG variants, it remains mostly indicative of structural alterations that may affect or not the channel function. We predict that this approach will be refined in the future by improving the analyses of the local structural impact of the variants and by increased coverage of the channel structure. Also, this approach needs validation on a larger set of variants by direct confrontation with experimental data from trafficking and biophysical properties.

As a result, it is clear that the index of the repolarisation power is the one that should be considered in priority to evaluate the pathogenicity of variants because it integrates all the possible deficiencies (biophysical ones and trafficking). The trafficking index offers a mechanistic evaluation of the cause for lack of channel functionality. The structural index, although less robust, offers a molecular explanation to trafficking and biophysical defects. One hope would be that the structural index would become predictive of a functional or a trafficking defect of a new variant not yet investigated. This may be relevant if clustering of variants can be encountered in structural channel hot spots.

### 5.3 How does the present study compare with other initiatives for high‐throughput characterisation of hERG variants?

There have been other initiatives for large‐scale investigation of hERG variants.^15^, ^16^, ^17^ One study elegantly used high‐throughput patch‐clamp to phenotype 23 variants in both homozygous and heterozygous conditions.^16^ The high‐throughput patch‐clamp system requires the production of stable cell lines for each variant and the use of the pIRES bicistronic plasmid to mimic heterozygous condition. The inequivalent expression of the two cassettes in bicistronic plasmids^18^ may lead to overestimation of the variant effect. In studies, like the present one, transient expression allowed equivalent expression of each transgene in the heterozygous condition. Nevertheless, this study reports the characterisation of the A561V variant with results consistent with ours since the peak tail current density was almost completely abolished, like in the case of our repolarisation power, and like an earlier study.^47^ Another high‐throughput study, used degenerate primers, a bar‐coded plasmid library, a hemagglutinin tag and flow cytometry to assess the effect of all possible variations of a pilot region of 11 residues on channel trafficking in homozygous situation.^17^ Although this technique provides an interesting and instrumental extensive map of trafficking defects of missense amino acid residues of the targeted region, it would require adaptation to the screening of specific clinically relevant variants and to the heterozygous condition. Here, again the A561V variant was investigated and consistently with our trafficking data there was a major reduction in trafficking (see Figure 2). In yet another large‐scale study, focusing mainly on trafficking issues by looking at glycosylation levels of hERG variants, three variants investigated in this study (C64Y, T74R and I96T) were giving consistent results with our own trafficking studies, highlighting the potential of the pHluorin tag for robust investigation of hERG variants.^15^

### 5.4 Perspectives and limitations of the present study

In this study, we have defined and optimised conditions for high‐throughput screening of hERG variants. This was a prerequisite step for handling hundreds of variants that have been listed in various databases. What would the follow‐up steps be? First, from our Cardiogen database, we will select the most clinically relevant hERG variants and generate a library of plasmids coding for these variants, using the Gibson assembly method described herein. Second, we will adapt the optimised biophysical protocol to an automated high‐throughput patch‐clamp system by taking into considerations the specificities of the automated patch‐clamp system. It will also be an opportunity to perform the recordings at 37°C, as mentioned above. Of note, the optimised protocol may need some adjustments to work at physiological temperature, namely with regard to step durations. Similarly, it is possible that adaptations may be required for variants with major alterations in the gating kinetics, a situation that we did not face with the selected variants used herein. Third, since the variants are most frequently only expressed by one allele, the pathogenicity of all the variants will be investigated in heterozygous conditions, similarly to what we have done here for four variants. One important technical issue to address will be the efficiency of transfection rate and success rate of cell recording using the automated patch‐clamp system. Establishing stable cell lines for each variant cannot be considered as a major improvement for fast phenotyping despite some reports.^16^ While in manual patch clamp, the cell to be recorded can be chosen, this is not the case for automated patch clamp which greatly diminishes the chances to record from a cell that is correctly transfected. This technical issue will need to be solved prior to a large‐scale investigation. Fourth, we have been working here with a pH‐sensitive tag for an accurate evaluation of hERG variant membrane expression, but, while we did not observe any impact of the tag presence on WT and D591H channel properties, we cannot fully exclude that this tag may influence the pathogenicity index of some given variants. For this reason, in future investigations, the tag will be used solely on the wild‐type hERG channel which will serve as a reporter for the eventual dominant‐negative effect of a given variant expressed without tag. For the patch‐clamp experiments, only non‐tagged hERG channels will be used to avoid any potential pitfall.

## CONFLICT OF INTEREST

The authors declare no conflict of interest.

## Supporting information

Supporting Information
