# Supplementary material for: A standardised hERG phenotyping pipeline to evaluate KCNH2 genetic variant pathogenicity
Source: Clin Transl Med. 2021 Nov 23;11(11):e609. doi: 10.1002/ctm2.609 (PMC8609418; doi:10.1002/ctm2.609)
Supplement: Supplementary file 1 — Supporting Information [file CTM2-11-e609-s001.pdf]

A standardized hERG phenotyping pipeline to evaluate *KCNH2* genetic variant pathogenicity

B.B.R. Oliveira Mendes[1], S. Feliciangeli[2], M. Ménard[1], F.C. Chatelain[2], M. Alameh[1], J. Montnach[1], S. Nicolas[1], B. Ollivier[1], J. Barc[1], I. Baró[1], J.J. Schott[1], V. Probst[3], F. Kyndt[3], I. Denjoy[4], F. Lesage[2], G. Loussouarn[1,§,*], M. De Waard[1,§,*]

Supplementary methods

Construction of hERG variants

An optimized hERG sequence fused to pHluorin tag (Opti-hERG-pHluorin), with inserted silent mutations for generation of specific enzyme restriction sites along the 3477 bp sequence, was synthesized by Genscript Biotech (NJ, USA) and subcloned into pcDNA5/FRT/TO vector (Invitrogen, MA, USA). We constructed 13 hERG variants with missense mutations by amplifying mutated overlapping fragments (Q5 Hot start High-Fidelity DNA polymerase M0493S, New England BioLabs, MA, USA) between the optimized restrictions sites and using the Gibson assembly strategy (NEBuilder HiFi DNA Assembly Master Mix E2621S, New England BioLabs, MA, USA).

For each construct, 4 primers were designed. Two external primers overlapped the sequences of two adjacent unique enzyme restriction sites and two internal primers, containing the mutation to be introduced, covered the mutated nucleotide sequence in the forward and reverse directions (***Table S1***). Two fragments were thus produced by PCR that overlapped in the region of the inserted mutation and at the ends to the two adjacent restriction enzymes. Using the plasmid template digested by the selected enzymes and the two mutated amplified fragments, the Gibson assembly process was used to produce the mutated plasmid. The plasmids were amplified using the NEB 10-beta competent *E. coli* bacterial strain (New England BioLabs, C3019H, MA, USA) and the sequences were confirmed by Sanger sequencing.

Cell culture

The Chinese Hamster Ovary cell line, CHO, was obtained from the American Type Culture Collection (CRL-11965) and cultured in Dulbecco's modified Eagle's medium (Gibco 41966-029, MA, USA) supplemented with 10% fetal calf serum (Eurobio, PAR, FR), 2 mM L-Glutamine and antibiotics (100 IU/mL penicillin and 100 µg/mL streptomycin, Corning, NY, EUA) at 5% CO_2, maintained at 37°C in a humidified incubator. This cell line was confirmed to be mycoplasma-free (MycoAlert, Lonza, CH).

Canis familiaris kidney cells (MDCK, ATCC CCL-34) were grown in 100-mm tissue-culture dishes (Falcon, Franklin Lakes, NJ) in Minimum Eagle's medium (Gibco, Life Technologies, Saint Aubin, FR) supplemented with 10% fetal calf serum (Hyclone, Thermo Fisher Scientific GMBH, Ulm, DE) and penicillin-streptomycin (Gibco, Life Technologies, Saint Aubin, FR) in a humidified incubator at 37°C (5% CO_2).

hERG plasmid transfection

The Fugene 6 transfection reagent (Promega, WI, USA) was used to transfect various hERG plasmids for manual patch-clamp experiments. Cells were cultured in 35-mm dishes at a 50% confluence and cotransfected, in homozygous condition with wild-type or mutant Opti-hERG-pHluorin (0.8 µg DNA) and eGFP (1.2 µg DNA) for fluorescence-based cell selection in microscopy. In heterozygous condition, cells were transfected with wild-type and mutant Opti-hERG-pHluorin (0.8 µg DNA each) and eGFP (0.4 µg DNA). Twenty-four hrs after transfection, cells were trypsinized, diluted (1/40) and replated on 35-mm dishes to obtain isolated cells for patch-clamp experiments. For channel membrane expression experiments in confocal microscopy, MDCK cells were grown on glass coverslips and transfected using Lipofectamine 2000 (Life technologies, Grand Island, NY, USA) according to the manufacturer's instructions.

Electrophysiology

One day after replating, transfected CHO cells were mounted on the stage of an inverted microscope and constantly perfused by a Tyrode solution maintained at 22.0 ± 2.0°C at a rate of 1-3 mL/min; HEPES-buffered Tyrode solution contained (in mmol/L): NaCl 145, KCl 4, $MgCl_2$ 1, $CaCl_2$ 1, HEPES 5, glucose 5, pH adjusted to 7.4 with NaOH. Patch pipettes (tip resistance: 2.0 to 2.5 MΩ) were pulled from soda lime glass capillaries (Kimble-Chase). The pipette was filled with intracellular medium containing (in mmol/L): KCl, 100; Kgluconate, 45; $MgCl_2$, 1; EGTA, 5; HEPES, 10; pH adjusted to 7.2 with KOH. Stimulation and data recording were performed with pClamp 10, an A/D converter (Digidata 1440A) and a Multiclamp 700B (all Molecular Devices) or VE-2 whole-cell patch clamp amplifier (Alembic Instruments). Currents were acquired in the whole-cell configuration, filtered at 10 kHz and recorded at a sampling rate of 20 kHz. Before series resistance compensation (70-80%), series of 20 30-ms steps to -80 mV were applied from HP of alternatively -70 mV and -90 mV to subsequently off-line calculate Cm and Rs values from the recorded currents. Protocols used are detailed in the figures.

Classical protocol analyses

For classical protocols, data were compiled and analyzed using ClampFit 10 (Axon Instruments), Microsoft Excel, and Prism (GraphPad Software, San Diego, CA). hERG activation and inactivation curves were obtained from the tail currents and fitted by Boltzmann equations. Current amplitudes for WT and all variants were extracted from the maximal value of the Boltzmann fit of the inactivation protocol.

Confocal microscopy

After 24 hr expression, MDCK cells observation was performed under an Ultraview Vox spinning disk confocal microscope from Perkin Elmer with a 60x/1.40 objective using a 488 nm laser and a 527/55 dichroic filter. Cells were imaged at 30 frames/min for 2 min under perfusion of extracellular buffer (140 NaCl, 5 KCl, 3 $MgCl_2$, 1 $CaCl_2$, 10 HEPES, pH 7.4 with NaOH). At 10 s the buffer was replaced by the same buffer in which HEPES is replaced with MES and titrated at pH 6.0 with NaOH (acidification medium) and switched back to the extracellular buffer after 20 s, and the cycle was repeated one more time. Signal intensity was measured, right before changing the buffer, under ImageJ using a home-made macro, and the results are expressed as the ratio of the difference between the fluorescence at pH 7.4 and the fluorescence at pH 6.0 over the fluorescence at pH 7.4 after subtraction of the background.
At the end of the observation, cells were treated with ionophores (nigericin and monensin both at 10 µM in extracellular buffer) to equilibrate the pH of all compartments with the incubation buffer. No increase of the signal could be observed with any of the constructs with ionophore-containing buffer at pH 7.4. On the opposite, treatment with ionophore-containing buffer at pH 6.0 caused a decrease of the fluorescence down to background level.

Statistical analyses

Data were expressed as mean ± SEM. Normality and equal variance were evaluated by Shapiro-Wilk and Levene's tests, respectively. Means were compared by Kruskal Wallis test or Two-way ANOVA. In case of multiple comparisons, a post hoc Dunn's or Dunnett's correction was used. Correlation analyses were indicated by r^2. Significance level was set at 0.05. All data were analyzed by GraphPad PRISM version 7.00 software (La Jolla, CA, USA).

Supplementary Table S1: Primers for hERG mutagenesis by Gibson assembly strategy.

hERG variant	*Nucleotide Mutation*	*Fragment*	*PCR Length bp*	*Primer*	*Primer sequence*	*%GC*	*Theorical TM*	*Reaction TM °C*	*Digested vector length*
R35W **classical mutagenese*	C -> T 103			Forward **1**	TCATCGCCAACGCTTGGGTGGAGAA	56%	75 °C	58 °C	
				Reverse **2**	GGCGATGATGAACTTACGGCTCTGGC	58%	74 °C		
C64Y	G -> A 191	HindIII to C64Y	237	Forward **1**	TCCGGACTCTAGCGTTTAAACTTAAG	42%	66 °C	66 °C	*Hind*III to *BamH*I 8995 bp
				Reverse **2**	AAGTCGCAGGTGTAGGGT	56%	66 °C		
		C64Y to BamHI	262	Forward **3**	TGCAGCGACCCTACACCT	61%	69 °C	69 °C	
				Reverse **4**	GTGGTTGGTGTCATGAGCCG	60%	69 °C		
T74R	C -> G 221	Hind to T74R	267	Forward **1**	TCCGGACTCTAGCGTTTAAACTTAAG	42%	66 °C	67	*Not*I to *Age*I 8777 pb
				Reverse **2**	GCGCGGCGCTGCCTGCGC	89%	83 °C		
		T74R to BamHI	233	Forward **3**	ACGGGCCGCGCAGGCAGC	83%	82 °C	67	
				Reverse **4**	GTGGTTGGTGTCATGAGCC	58%	66 °C		
K93E	A -> G 277	HindIII to K93E	323	Forward **1**	TCCGGACTCTAGCGTTTAAACTTAAG	42%	66 °C	67 °C	*Hind*III to *BamH*I 8995 bp
				Reverse **2**	CGATTTCCACTTCGCGCTC	58%	67 °C		
		K93E to BamHI	177	Forward **3**	CCGAGGAGCGCGAAGTG	71%	69 °C	67 °C	
				Reverse **4**	GTGGTTGGTGTCATGAGCC	58%	66 °C		
I96T	T -> C	HindIII to I96T	333	Forward **1**	TCCGGACTCTAGCGTTTAAACTTAAG	42%	66 °C	67 °C	
				Reverse **2**	CGGTAGAAGGCGGTTTCC	61%	66 °C		

	287	I96T to BamHI	167	Forward 3	GCAAAGTGGAAACCGCCTTC	55%	67 °C	67 °C	*Hind*III to *BamH*I 8995 bp
				Reverse 4	GTGGTTGGTGTCATGAGCC	58%	66 °C		
R176W	C -> T 526	BamHI to R176W	147	Forward 1	TGGTGATGGAGAAGGACATGGT	50%	68 °C	69 °C	*BamH*I to *Kpn*I 8862 pb
				Reverse 2	GACGACTCCCAGGCCGT	69%	70 °C		
		R176W to KpnI	487	Forward 3	GCTGACGGCCTGGGAG	75%	69 °C	70°C	
				Reverse 4	GGAATCTTGCTAATGGTGCGGTACC	52%	70 °C		
R328C	C -> T 982	BamHI to R328C		Forward 1	TGGTGATGGAGAAGGACATG	50%	64 °C	63 °C	*BamH*I to *Nsi*I 8144 bp
				Reverse 2	TGCTAATGGTGCAGTACC	50%	62 °C		
		R328C to NsiI		Forward 3	TCGTGCGGTACTGCACCA	61%	70 °C	70 °C	
				Reverse 4	GTTGCCGATGGCGTACCAG	63%	69 °C		
R534C	C -> T 1600	KpnI to R534C	659	Forward 1	CACCTCGGACTCCGACCTC	68%	70 °C	70 °C	*Kpn*I to *Nsi*I 8710 pb
				Reverse 2	GCGCCACGCACACCA	73%	71 °C		
		R534C to NsiI	132	Forward 3	TGCGGCTGGTGTGCGT	69%	72 °C	70 °C	
				Reverse 4	GTTGCCGATGGCGTACCAG	63%	69 °C		
A561V **classical mutagenese*	C -> T 1682			Forward 1	GCGCTCATCGTGCACTGGCTA	62%	71°C	68°C	
				Reverse 2	AAAGGTGCACATGAGCAAGAACAG	46%	67°C		
G584S	G -> A 1750	KpnI to G584S	809	Forward 1	CACCTCGGACTCCGACCTC	68%	70 °C	70 °C	*Kpn*I to *BsiW*I 7721 pb
				Reverse 2	TGTGCAGCCAGCTGATGC	61%	69 °C		
			967	Forward 3	GACTCACGCATCAGCTGG	61%	66 °C	67 °C	

		G584S to BsiWI		Reverse 4	GCTGCTCCGTGTCCTTGTC	63%	69 °C		
D591H	G -> C 1771	KpnI to D591H	830	Forward 1	CACCTCGGACTCCGACCTC	68%	70 °C	71 °C	*Kpn*I to *BsiWI* 7725 pb
				Reverse 2	TGCCTATCTGGTGGCCCAG	63%	70 °C		
		D591H to BsiWI	948	Forward 3	CACAACCTGGGCCACCAG	67%	70 °C	70 °C	
				Reverse 4	GCTGCTCCGTGTCCTTGTC	63%	69 °C		
R835P	G -> C 2504	NsiI to R835P	841	Forward 1	TCATCGCGCACTGGCTAG	61%	68 °C	60 °C	*NsiI to BsiWI* 8439 bp
				Reverse 2	AGCAGGTCGTCCGGATGGAT	60%	71 °C		
		R835P to BsiWI	213	Forward 3	ACAAGATCCATCCGGACGACC	57%	69 °C	70 °C	
				Reverse 4	GCTGCTCCGTGTCCTTGTC	63%	69 °C		
P1026L	C -> T 3077	BsiWI to P1026L	432	Forward 1	GCGCAAGTTGTCCTTCCG	61%	67 °C	67 °C	*BsiWI* to *Age*I 8442 pb
				Reverse 2	GGCTGGAGAGGAGGATGT	61%	66 °C		
		P1026L to AgeI	623	Forward 3	TCCTCAACATCCTCCTCTC	53%	63 °C	64 °C	
				Reverse 4	TAATTCAACAAGAATTGGGACAACAC	35%	63 °C		

Supplementary Table S2: Comparison between hERG properties in the presence and absence of the pHluorin tag using the optimized protocol. For peak tail current densities, cells lacking current in the pHluorin condition were included (such cells were not observed in the condition without pHluorin). There was no significant difference for the biophysical parameters. Mean ± SD.

	**WT Opti-hERG-pHluorin**	**WT Opti-hERG**	**D591H Opti-hERG-pHluorin**	**D591H Opti-hERG**
**Peak tail current**	256.8 ± 218.6 pA/pF (25)	531.9 ± 316.8 pA/pF (26)	199.9 ± 224.7 pA/pF (19)	266.3 ± 152.4 pA/pF (12)
**half-activation potential**	-19.9 ± 7.0 mV (14)	-19.2 ± 4.2 mV (22)	-15.3 ± 4.1 mV (10)	-19.0 ± 8.4 mV (11)
**activation slope**	11.2 ± 1.7 mV (14)	10.6 ± 1.9 mV (22)	10.4 ± 1.5 mV (10)	10.9 ± 2.4 mV (11)
**half-inactivation potential**	-83.3 ± 6.0 mV (15)	-81.0 ± 4.6 mV (23)	-59.7 ± 10.0 mV (11)	-59.1 ± 13.6 mV (11)
**inactivation slope**	21.5 ± 1.7 mV (15)	21.6 ± 1.9 mV (23)	20.0 ± 5.2 mV (11)	21.1 ± 5.2 mV (11)

Supplementary Table S3: Mean ± SD for the parameters of WT and hERG channel variants analyzed in homozygous conditions. Number of recorded cells are in parentheses.

			PAS					N-linker		VSD	Pore			CNBHD	C-tail	
		**WT**	**R35W**	**C64Y**	**T74R**	**K93E**	**I96T**	**R176W**	**R328C**	**R534C**	**A561V**	**G584S**	**D591H**	**R835P**	**P1026L**	**GFP**
	**Fluorescence variation**	35.1 ± 14.9 (18)	32.2 ± 13.5 (18)	15.5 ± 9.9 (14)	10.3 ± 2.5 (10)	26.6 ± 13.7 (14)	14.3 ± 13.7 (13)	9.2 ± 3.5 (12)	19.4 ± 8.9 (18)	10.6 ± 4.7 (13)	14.3 ± 7.2 (15)	21.3 ± 10.1 (12)	28.0 ± 14.1 (22)	12.4 ± 14.2 (18)	6.6 ± 3.5 (14)	NA
**Classical protocols Mean ± SD**	**Current density (pA/pF)**	152 ± 163 (163)	105 ± 108 (19)	13.5 ± 38.4 (17)	0.0 ± 0.0 (20)	91.1 ± 147 (22)	5.0 ± 15.2 (28)	41.6 ± 68.5 (37)	146 ± 156 (33)	0.0 ± 0.0 (12)	0.0 ± 0.0 (21)	39 ± 48 (33)	158 ± 218 (36)	0.0 ± 0.0 (13)	31.3 ± 77.6 (21)	0.0 ± 0.0 (27)
	**Half-activation potential (mV)**	-18.2 ± 7.1 (78)	-16.3 ± 5.3 (11)	NA	NA	-14.9 ± 6.5 (10)	NA	-26.1 ± 4.9 (11)	-15.3 ± 3.1 (14)	NA	NA	-18.1 ± 7.9 (10)	-19.9 ± 6.2 (20)	NA	NA	NA
	**Slope of activation (mV)**	7.4 ± 1.2 (78)	6.5 ± 1.0 (11)	NA	NA	7.8 ± 1.8 (10)	NA	7.0 ± 1.6 (11)	7.7 ± 1.3 (14)	NA	NA	6.7 ± 1.1 (10)	7.7 ± 1.1 (20)	NA	NA	NA
	**Half-inactivation potential (mV)**	-86.2 ± 7.4 (101)	-85 ± 11 (11)	NA	NA	-79.4 ± 10.4 (10)	NA	-86 ± 4 (11)	-83.5 ± 5.6 (16)	NA	NA	-94 ± 5 (12)	-66 ± 8 (22)	NA	NA	NA
	**Slope of inactivation (mV)**	20.1 ± 2.6 (101)	21.9 ± 4.1 (11)	NA	NA	20.7 ± 3.5 (10)	NA	19.9 ± 1.7 (11)	20.4 ± 2.2 (16)	NA	NA	19.6 ± 1.8 (12)	23.4 ± 4.8 (22)	NA	NA	NA
**Optimized protocol Mean ± SD**	**Repolarization power**	1.0 ± 1.0 (140)	0.5 ± 0.7 (35)	0.2 ± 0.3 (12)	0.1 ± 0.1 (21)	1.0 ± 1.3 (38)	0.1 ± 0.1 (26)	0.3 ± 0.3 (30)	0.5 ± 0.6 (47)	0.1 ± 0.1 (11)	0.1 ± 0.1 (19)	0.2 ± 0.1 (37)	2.6 ± 3.9 (41)	0.3 ± 0.6 (14)	0.2 ± 0.4 (25)	0.1 ± 0.1 (33)
	**Current density (pA/pF)**	204 ± 235 (123)	94 ± 149 (35)	29.7 ± 76.9 (14)	0.0 ± 0.0 (21)	164 ± 195 (32)	4.2 ± 14.5 (25)	42 ± 66 (26)	109 ± 145 (42)	0.0 ± 0.0 (11)	0.4 ± 1.7 (19)	62 ± 87 (35)	192 ± 268 (31)	0.0 ± 0.0 (14)	23 ± 85 (24)	0.0 ± 0.0 (33)
	**Half-activation potential (mV)**	-19.5 ± 10.2 (75)	-18.4 ± 6.1 (11)	NA	NA	-19.2 ± 10.2 (16)	NA	-23.9 ± 5.4 (10)	-17.7 ± 4.7 (13)	NA	NA	-19.4 ± 11.1 (10)	-18.7 ± 8.8 (14)	NA	NA	NA
	**Slope of activation (mV)**	11.6 ± 2.2 (75)	11.3 ± 2.7 (11)	NA	NA	9.3 ± 2.3 (16)	NA	14.2 ± 3.6 (10)	12.6 ± 2.3 (13)	NA	NA	9.5 ± 1.6 (10)	11.5 ± 2.5 (14)	NA	NA	NA
	**Half-inactivation potential (mV)**	-84 ± 7 (75)	-78 ± 7 (11)	NA	NA	-81 ± 8 (16)	NA	-86 ± 5 (11)	-87 ± 6 (17)	NA	NA	-93 ± 5 (10)	-67 ± 12 (15)	NA	NA	NA
	**Slope of inactivation (mV)**	20.3 ± 2.6 (75)	21.1 ± 2.8 (11)	NA	NA	21.9 ± 3.3 (16)	NA	22.8 ± 2.5 (11)	20.9 ± 4.3 (17)	NA	NA	20.6 ± 2.1 (10)	22.1 ± 4.4 (15)	NA	NA	NA

Supplementary Figure S1. hERG structure and Opti-hERG-pHluorin plasmid. (**A**) Structure of hERG channel in its functional tetrameric form. The Pore domain, Voltage sensor domain (VSD), CNBHD domain, and PAS domain are visible in this modelling. (**B**) Representation of the pCDNA5/FRT/TO Opti-hERG-pHluorin plasmid template with the silent restriction sites included in the sequence encoding hERG (*Bam*HI, *Kpn*I, *Nsi*I, *Bsi*WI and *Not*I) represented by the red arrows (►).CG rich regions are located between *Bam*HI – *Kpn*I and *BsiW*I – *Not*I. The plasmid has a CMV promoter and an ampicillin resistance gene. The tag sequence was added 3' of the hERG nucleotide sequence that encodes the transmembrane segment of transferrin followed by the fluorescent pHluorin protein. hERG encoding sequence counts 1159 amino acids. The different domains of the channel are represented, each with its appropriate amino acid numbering. Thirteen plasmids were generated each carrying one of the mutations represented by the black arrows (►) in different domains of hERG channel.

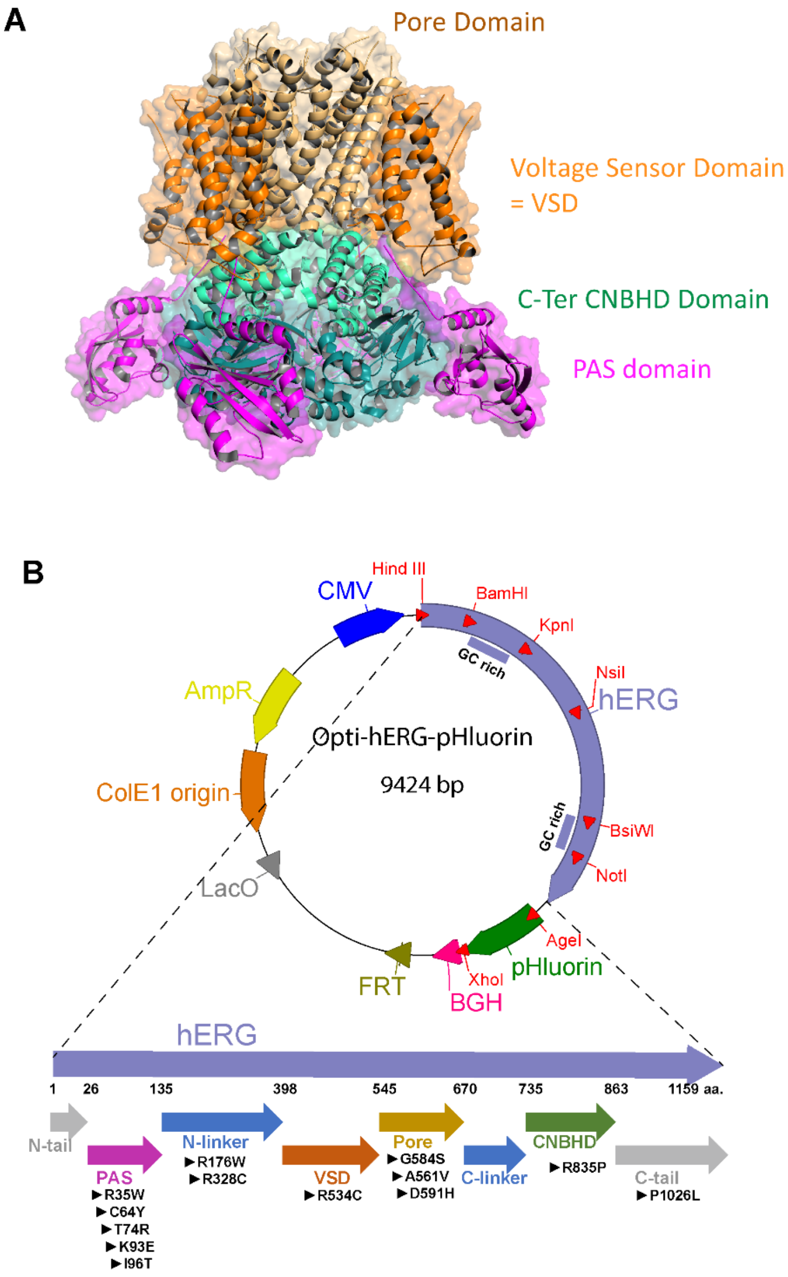

Supplementary Figure S2: Comparison of WT and D591H hERG properties in presence and absence of pHluorin tag. (**A**) Violin plots of the time integral of the current density (repolarization power) of WT and D591H hERG variant. **p value <0.01, ns non-significant. Mann-Whitney tests. (**B**) Scattered plots of WT and D591H hERG variant maximal current densities extracted from the inactivation protocol SP3. (**C**) and (**D**) Tukey plots of WT and D591H hERG variant half-activation potential and activation slope, respectively. Black dots represent individual values. (**E**) and (**F**) Tukey plots of WT and D591H hERG variant half-inactivation potential and inactivation slope, respectively. Black dots represent individual values. See Figure S4 and text for details on the optimized protocol SP3 and repolarization power.

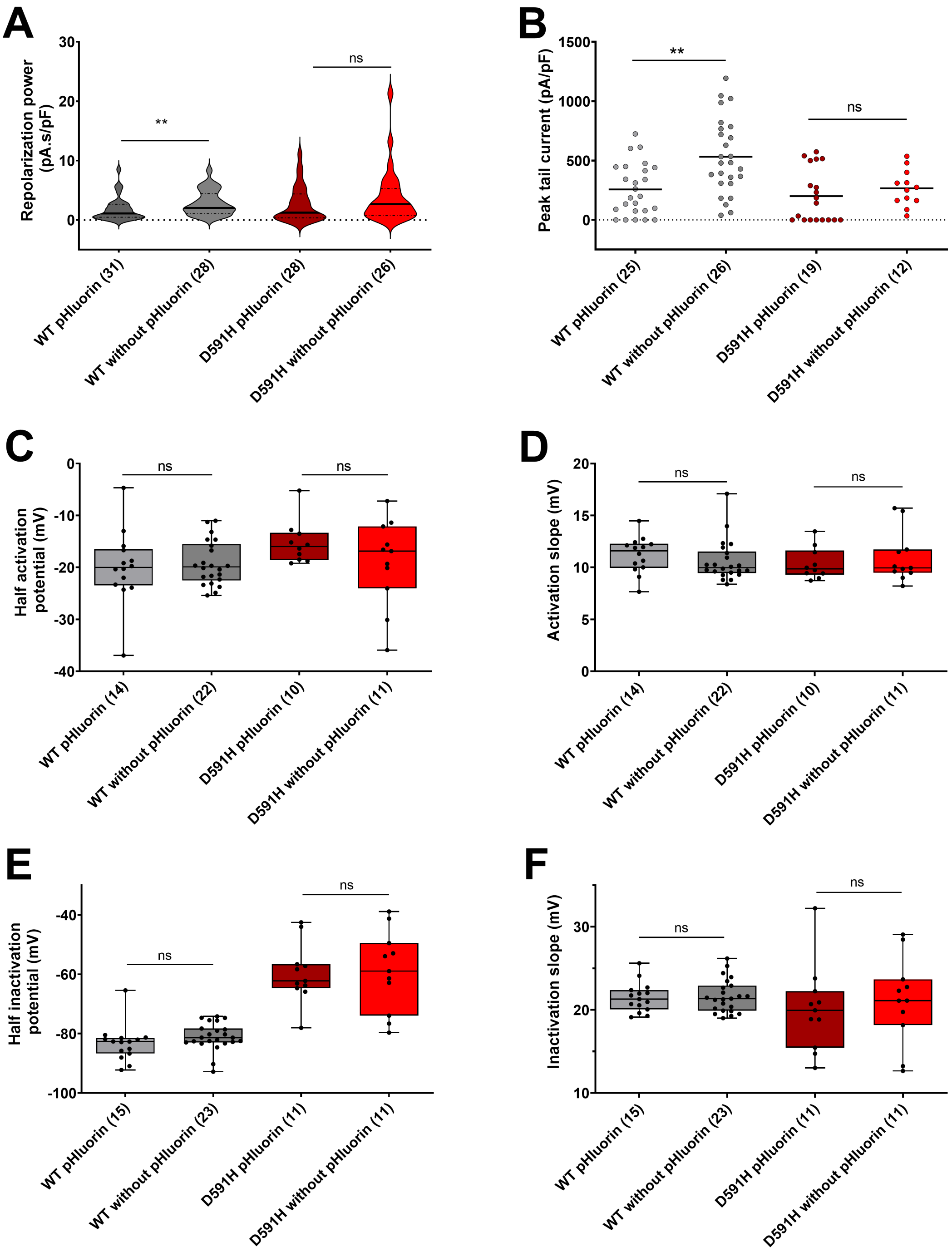

Supplementary Figure S3: Comparison of WT hERG properties between the batches used during the course of the study. The constancy in hERG activation and inactivation properties were evaluated in 4 batches throughout the acquisition process of the variants studied in conventional patch-clamp. No statistical difference was observed between the 4 batches analyzed. (**A**) Violin plots of the 4 WT hERG batches maximal current densities extracted from the inactivation protocol (Figure 1B). Non-significant p values, Kruskal Wallis test. A dotted line crosses the median of the WT. (**B**) Activation curves of the 4 WT hERG batches, obtained from tail currents using the protocol shown in Figure 1A, lines are Boltzmann fits to the data. (**C**) and (**D**) Tukey plots of the 4 WT hERG batches half-activation potential and slope, respectively. Non-significance p value, Kruskal Wallis test. (**E**) Inactivation curves of the 4 WT hERG batches, obtained from tail currents using the protocol shown in Figure 1B, lines are Boltzmann fits to the data. (**F**) and (**G**) Tukey plots of the 4 WT hERG batches half-activation potential and slope, respectively. Non-significance p value, Kruskal Wallis test.

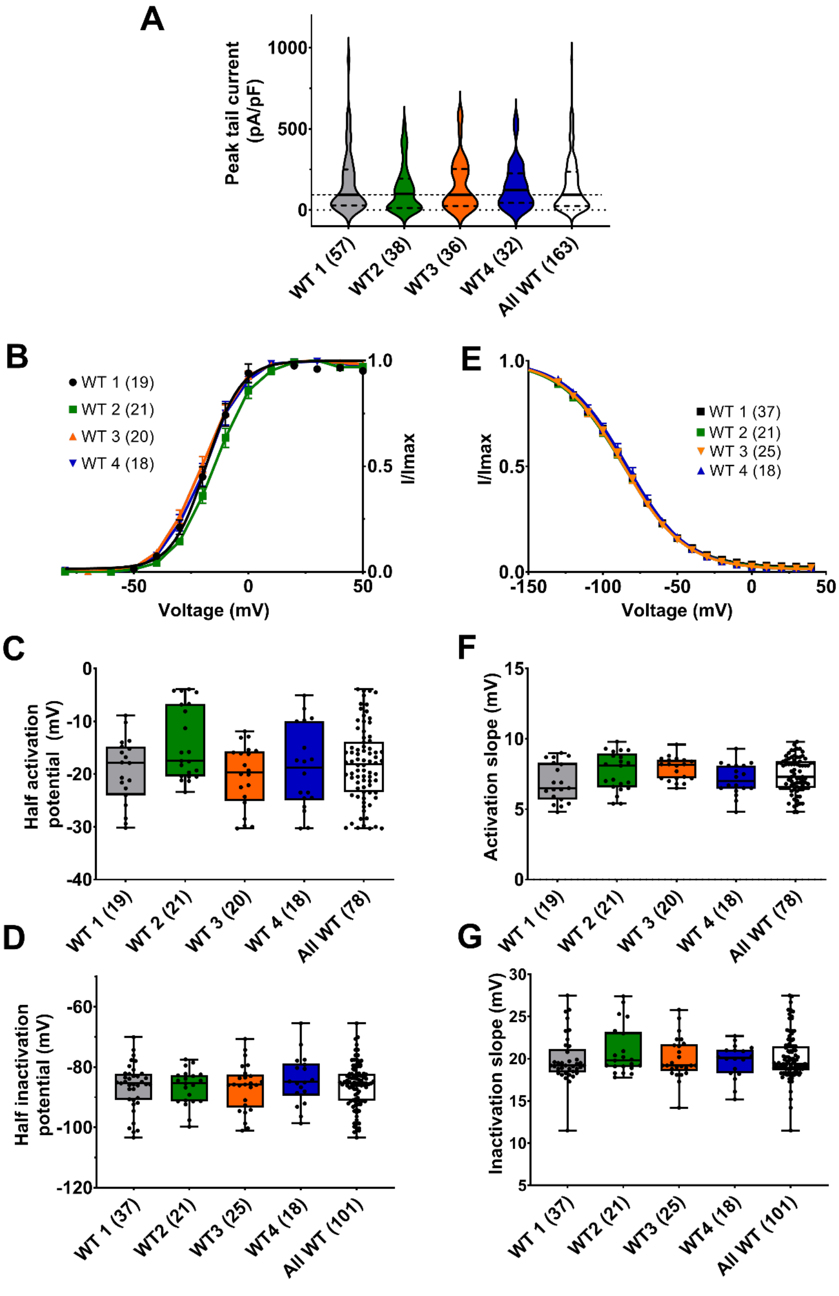

Supplementary Figure S4. A new optimized voltage-clamp protocol for complete and fast-track extraction of hERG channel parameters. The 35-second long protocol is divided in 7 subprotocols (SP). (**SP1**) The voltage protocol used is generated using the O'Hara and Rudy model of sub-epicardial ventricular action potential [1] at a cycle length of 1 sec, corrected with the liquid junction potential. (**SP2**) Steady-state activation voltage protocol with depolarization steps of optimized durations, longer for low voltages and shorter for high voltages. (**SP3**) Steady-state inactivation voltage protocol with a single activation step to fully open the activation gates before evaluating inactivation per se. (**SP4**) Deactivation kinetics: Deactivation time constants were extracted at -120, -110 and -100 mV. (**SP5**) Inactivation kinetics, studied after channel activation at +70 mV. (**SP6**) Recovery from inactivation kinetics, studied after channel activation at +70 mV. (**SP7**) Activation kinetics: Activation kinetics at 0 and +70 mV obtained by repeated activation pulses to 0/+70 mV, separated by 10-ms step to -100 mV to deinactivate the channels and reveal the extent of cumulated hERG channel activation. Peak currents were measured for the activated currents at 0 or +70 mV.

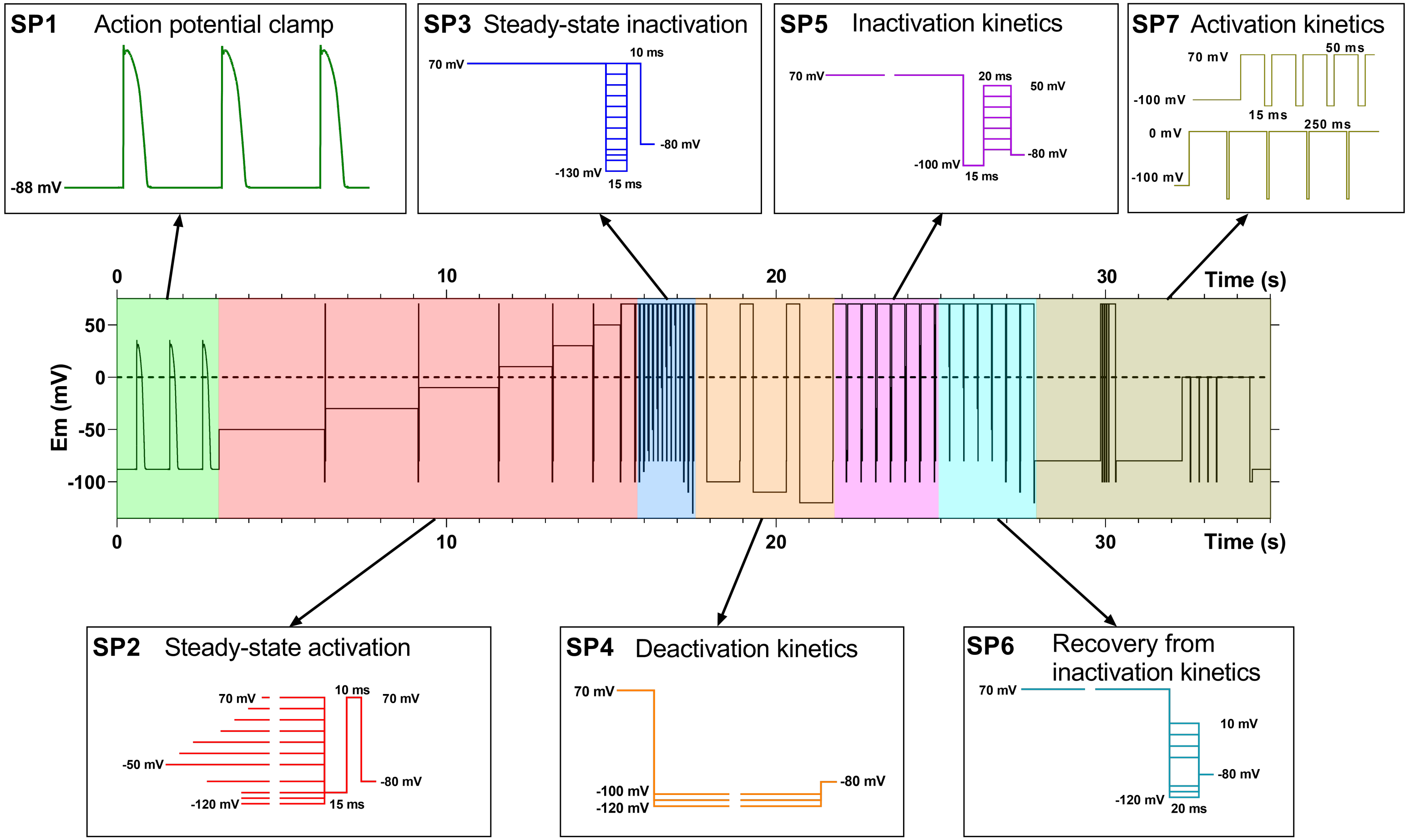

Supplementary Figure S5: Acquisition file generated by R automated routine of voltage-clamp optimized subprotocols analyses **(SP1, SP2, SP3, SP4, SP5, SP6, SP7)**

SP1

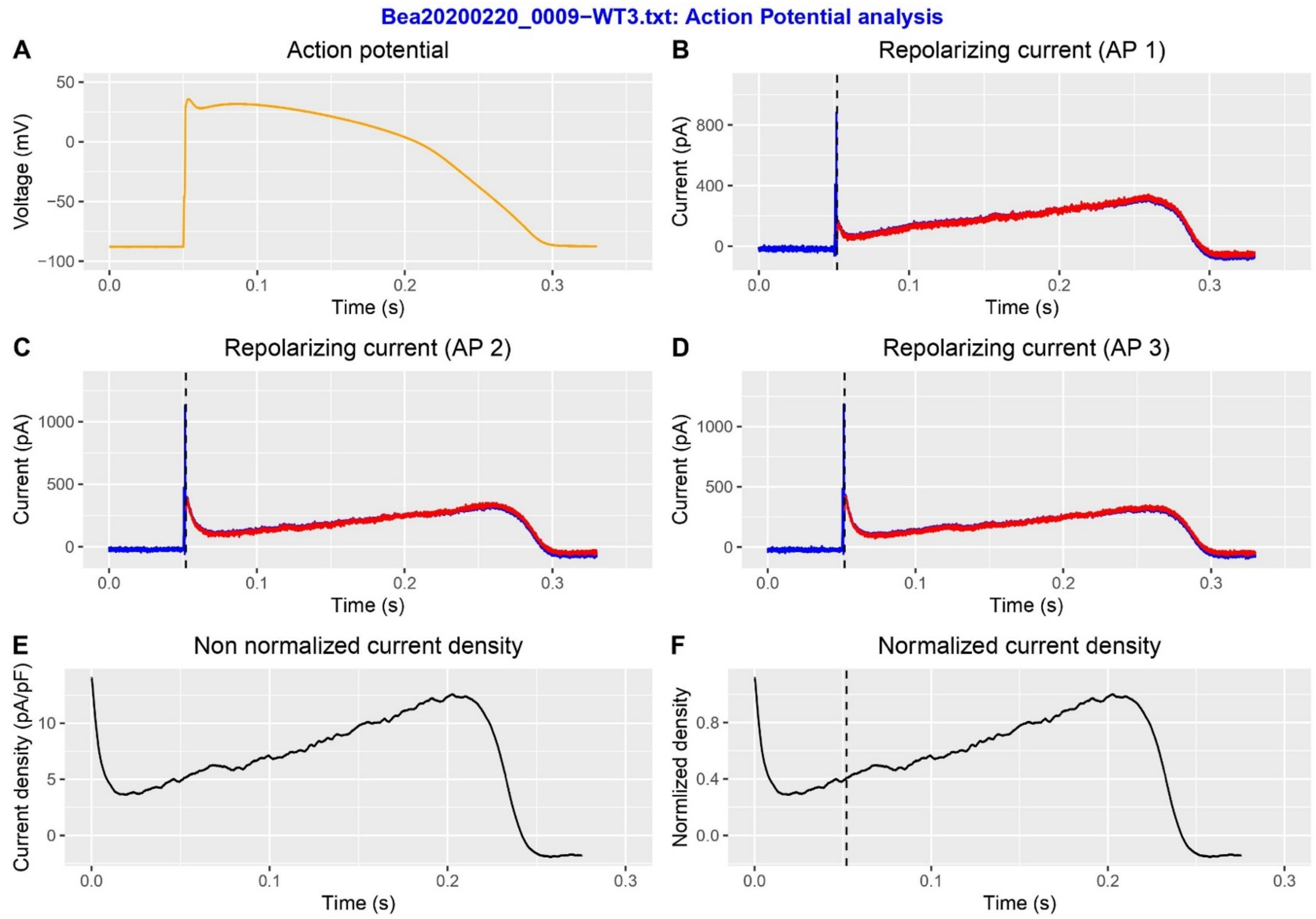

SP2

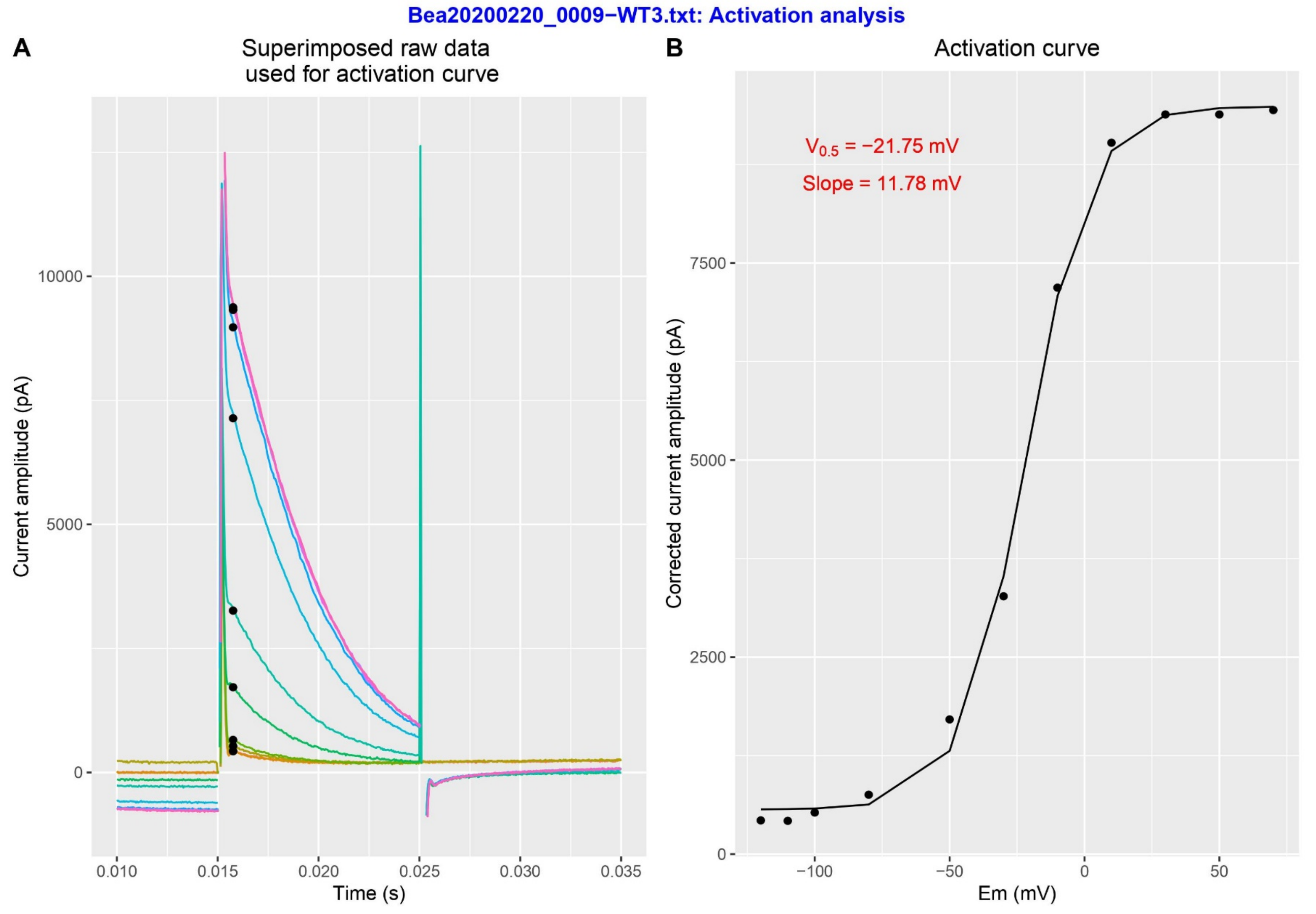
Bea20200220_0009−WT3.txt: Activation analysis
A
Superimposed raw data
used for activation curve
Current amplitude (pA)
10000
5000
0
0.010
0.015
0.020
0.025
0.030
0.035
Time (s)
B
Activation curve
V0.5 = −21.75 mV
Slope = 11.78 mV
Corrected current amplitude (pA)
7500
5000
2500
0
−100
−50
0
50
Em (mV)

SP3

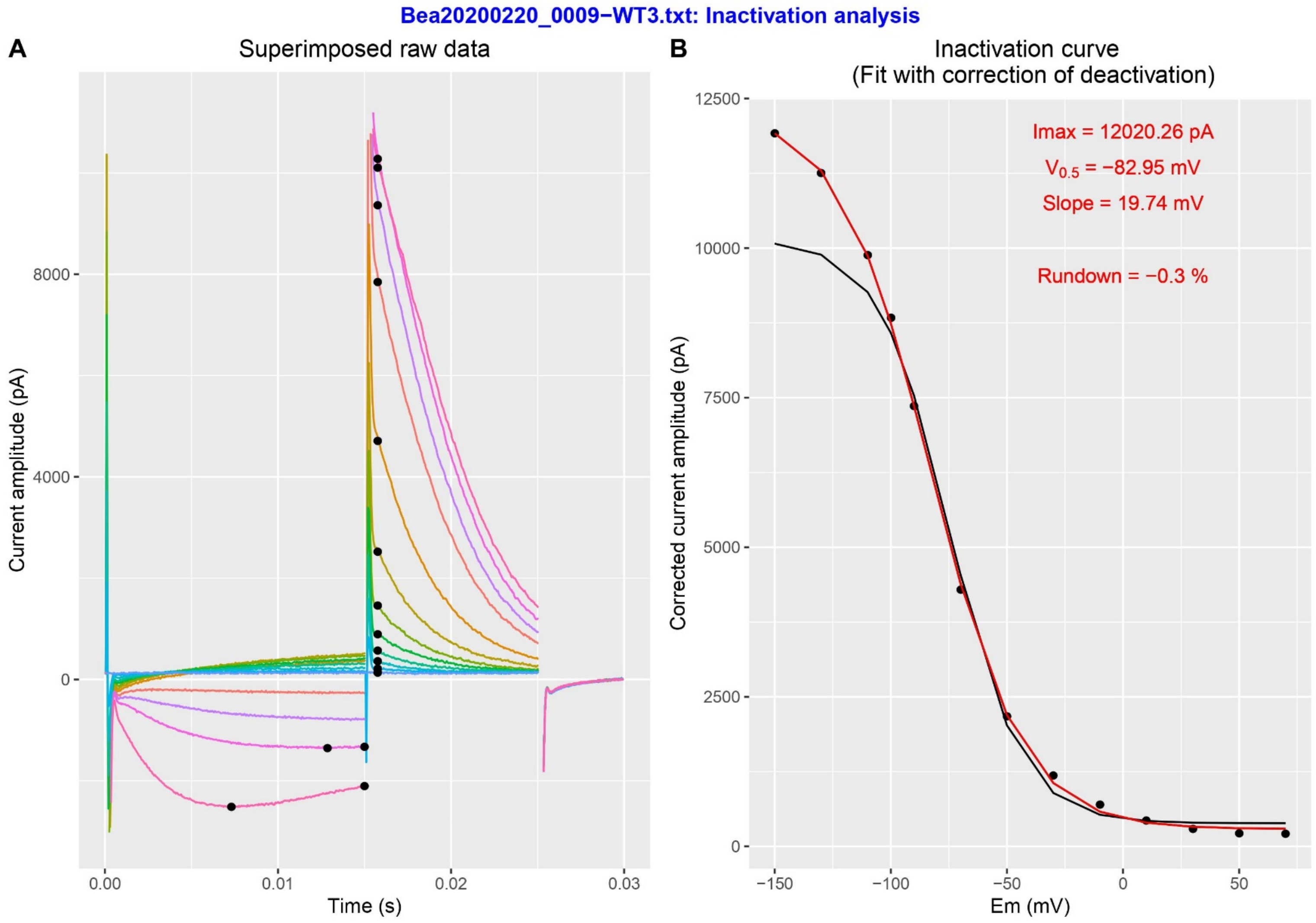
Bea20200220_0009−WT3.txt: Inactivation analysis
A
Superimposed raw data
Current amplitude (pA)
8000
4000
0
0.00
0.01
0.02
0.03
Time (s)
B
Inactivation curve
(Fit with correction of deactivation)
Corrected current amplitude (pA)
12500
10000
7500
5000
2500
0
−150
−100
−50
0
50
Em (mV)
Imax = 12020.26 pA
V0.5 = −82.95 mV
Slope = 19.74 mV
Rundown = −0.3 %

SP4

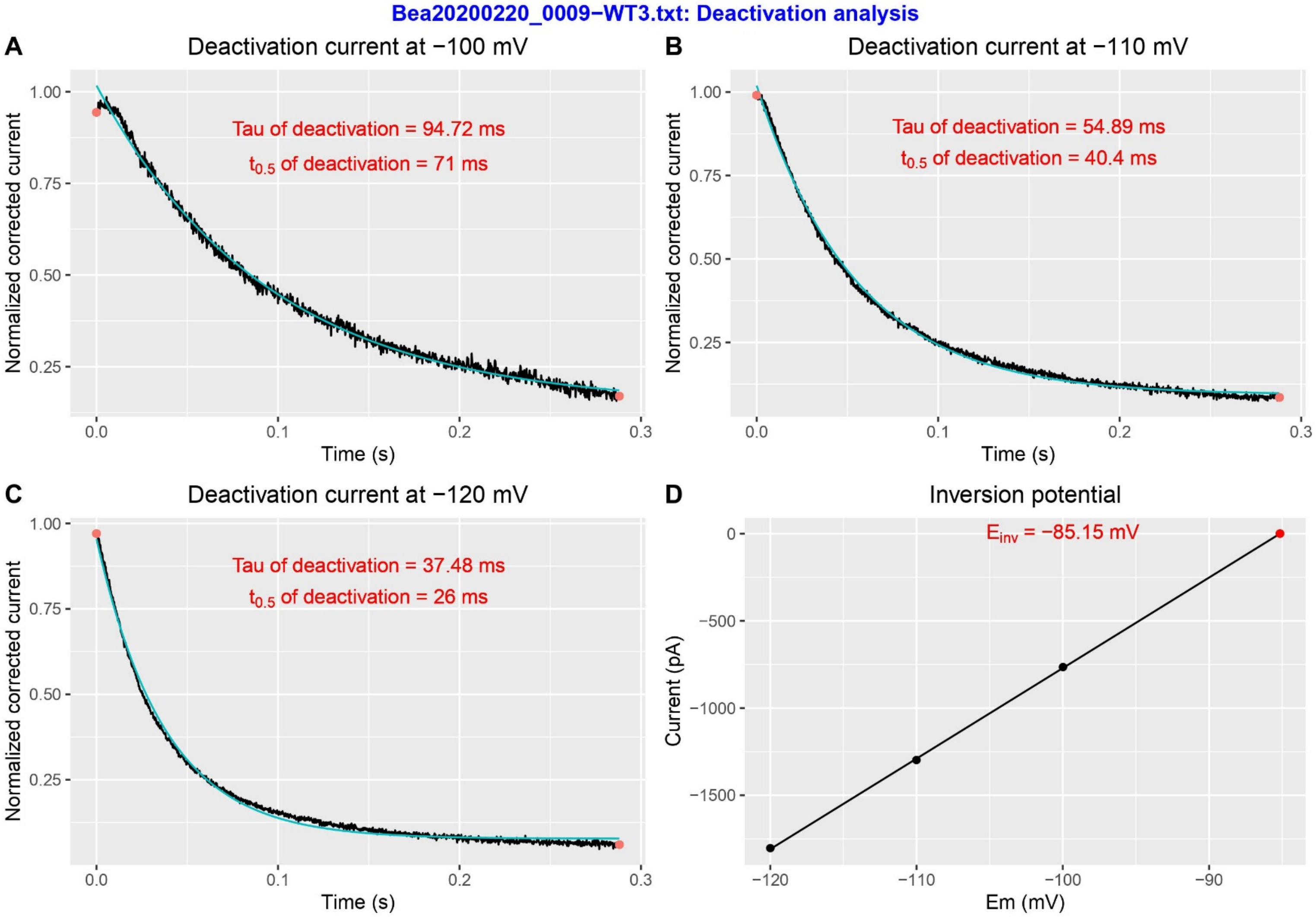
Bea20200220_0009−WT3.txt: Deactivation analysis
A
Deactivation current at −100 mV
Tau of deactivation = 94.72 ms
t0.5 of deactivation = 71 ms
Normalized corrected current
1.00
0.75
0.50
0.25
0.0
0.1
0.2
0.3
Time (s)
B
Deactivation current at −110 mV
Tau of deactivation = 54.89 ms
t0.5 of deactivation = 40.4 ms
Normalized corrected current
Time (s)
C
Deactivation current at −120 mV
Tau of deactivation = 37.48 ms
t0.5 of deactivation = 26 ms
Normalized corrected current
Time (s)
D
Inversion potential
Einv = −85.15 mV
Current (pA)
0
−500
−1000
−1500
−120
−110
−100
−90
Em (mV)

SP5

SP6

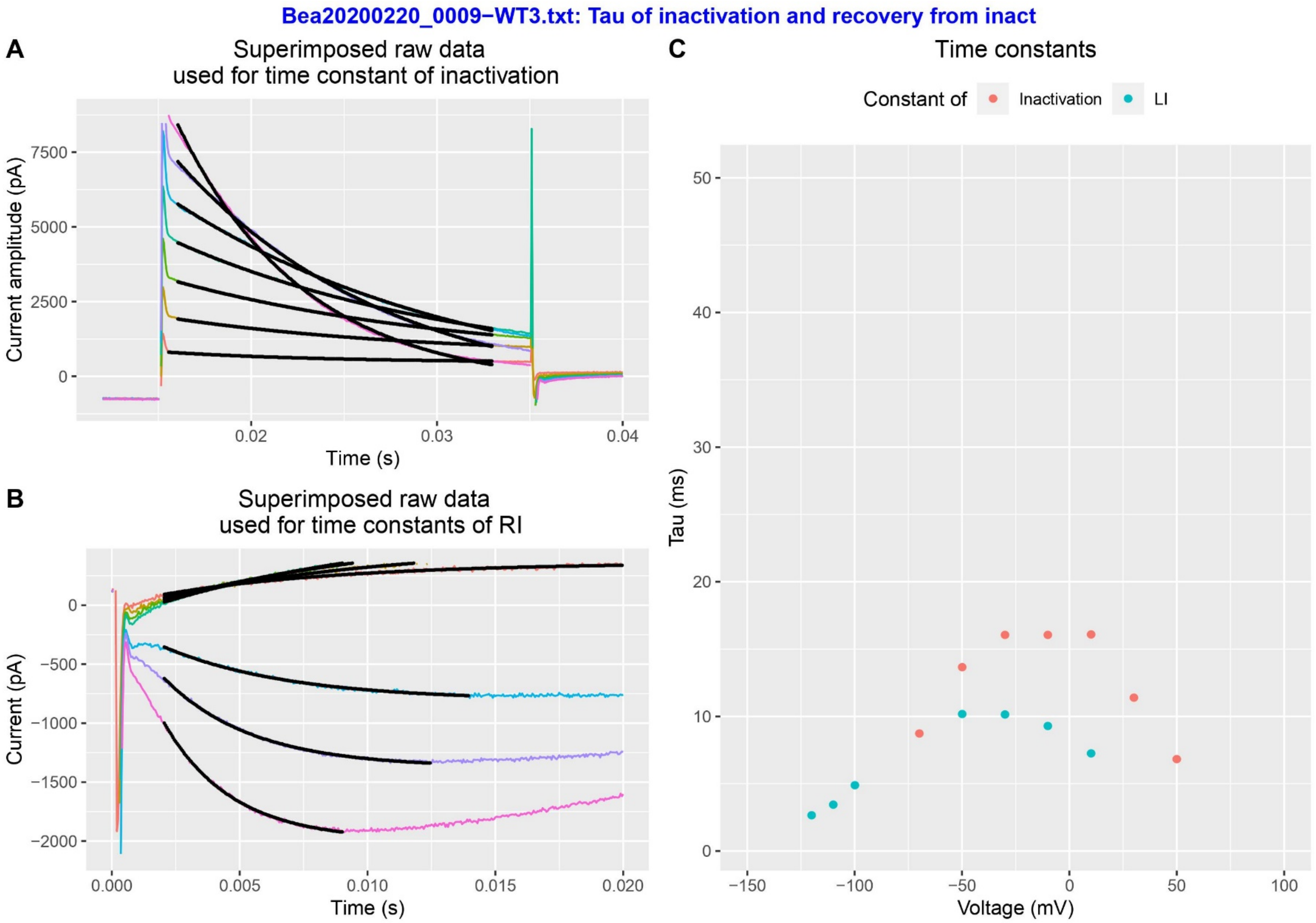
Bea20200220_0009−WT3.txt: Tau of inactivation and recovery from inact
A
Superimposed raw data
used for time constant of inactivation
Current amplitude (pA)
Time (s)
B
Superimposed raw data
used for time constants of RI
Current (pA)
Time (s)
C
Time constants
Constant of
Inactivation
LI
Tau (ms)
Voltage (mV)

SP7

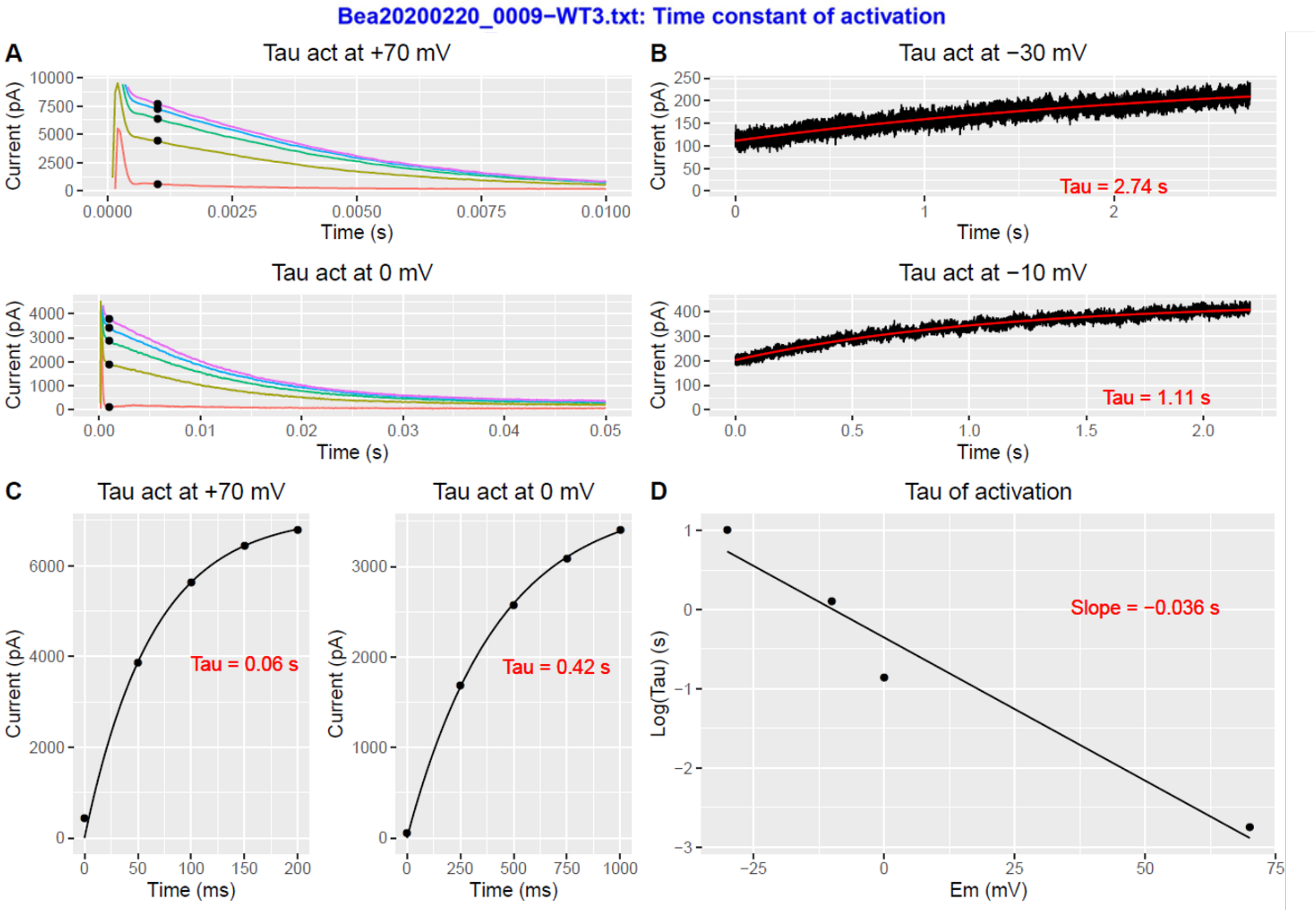

Bea20200220_0009−WT3.txt: Time constant of activation
A
Tau act at +70 mV
Current (pA)
Time (s)
Tau act at 0 mV
B
Tau act at −30 mV
Tau = 2.74 s
Tau act at −10 mV
Tau = 1.11 s
C
Tau act at +70 mV
Tau = 0.06 s
Time (ms)
Tau act at 0 mV
Tau = 0.42 s
D
Tau of activation
Slope = −0.036 s
Log(Tau) (s)
Em (mV)

Supplementary Figure S6. Test of negative dominance on function of four new hERG variants lacking activity in homozygous conditions. (**A**) Mean ± sem current density recordings of WT and four variants in heterozygous conditions during action potential clamp. (**B**) Violin plots of the time integral of the current density as shown in (**A**) called repolarization power for WT and the four hERG variants. **p value <0.01, *p value < 0.05, ns non-significant. Mann-Whitney tests. (**C**) Comparison of activation curves of WT and four hERG variants in heterozygous conditions obtained from tail currents using the SP2 protocol. Lines are Boltzmann fits to the data. (**D**) Comparison of inactivation curves of WT and four hERG variants in heterozygous conditions obtained from tail currents using the SP3 protocol. Lines are Boltzmann fits to the data. (**E**) and (**F**) Tukey plots of half-activation potential and activation slope, respectively, for WT and four hERG variants in heterozygous conditions. Black dots represent individual values. (**G**) and (**H**) Tukey plots of half-inactivation potential and inactivation slope, respectively, for WT and four hERG variants in heterozygous conditions. Black dots represent individual values.

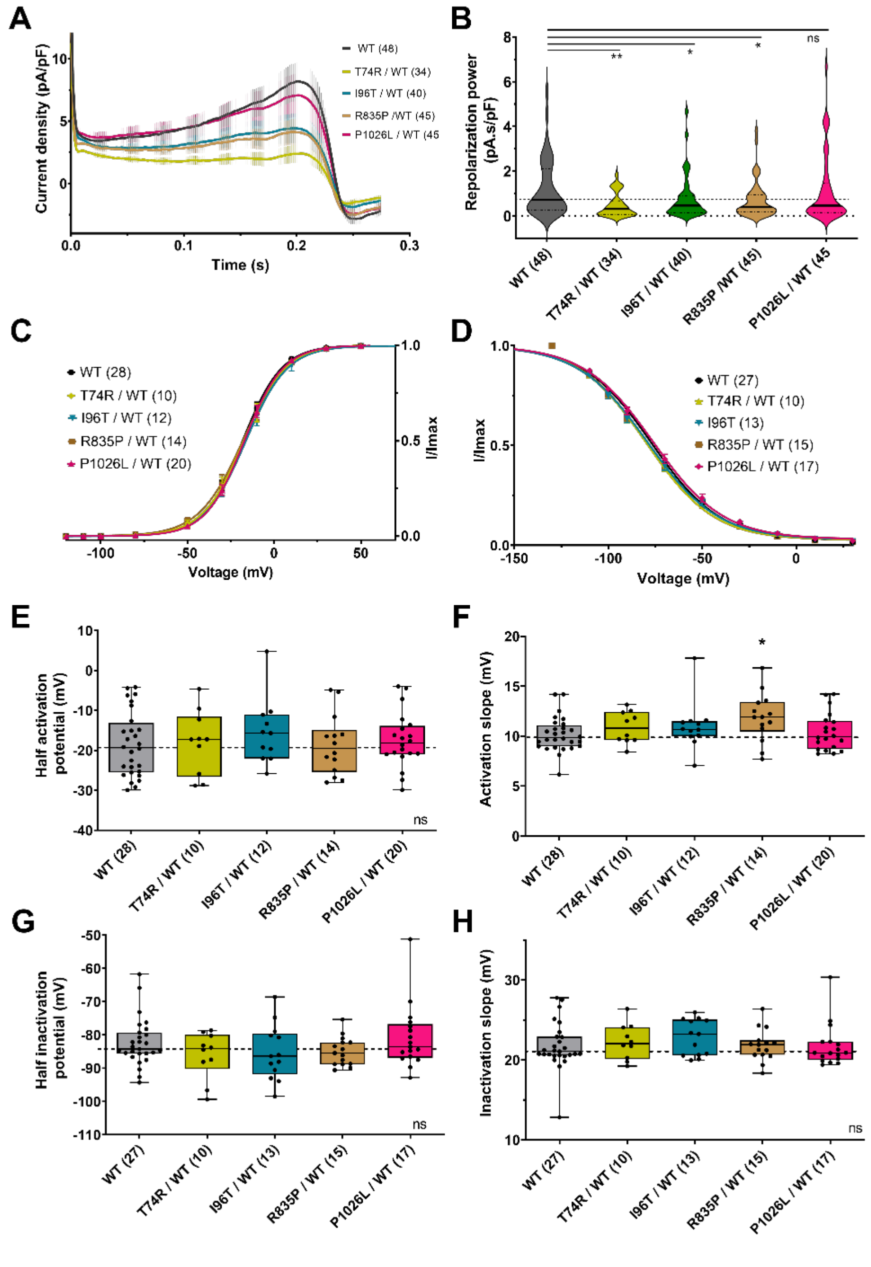

Supplementary Figure S7. Kinetic parameters extracted from the optimized protocol for WT and four hERG variants in heterozygous conditions. Kinetics subprotocols used (SP4-SP7) are presented in Figure S4. (**A**) Mean ± sem of activation tau for several membrane potentials. ns, non-significant. Mann-Whitney test. (**B**) Mean ± sem of deactivation $T_{0.5}$ for several membrane potentials. (**C**) Mean ± sem of inactivation tau for several membrane potentials. *p value vs. WT hERG <0.05. (**D**) Mean ± sem of recovery from inactivation tau for several membrane potentials.

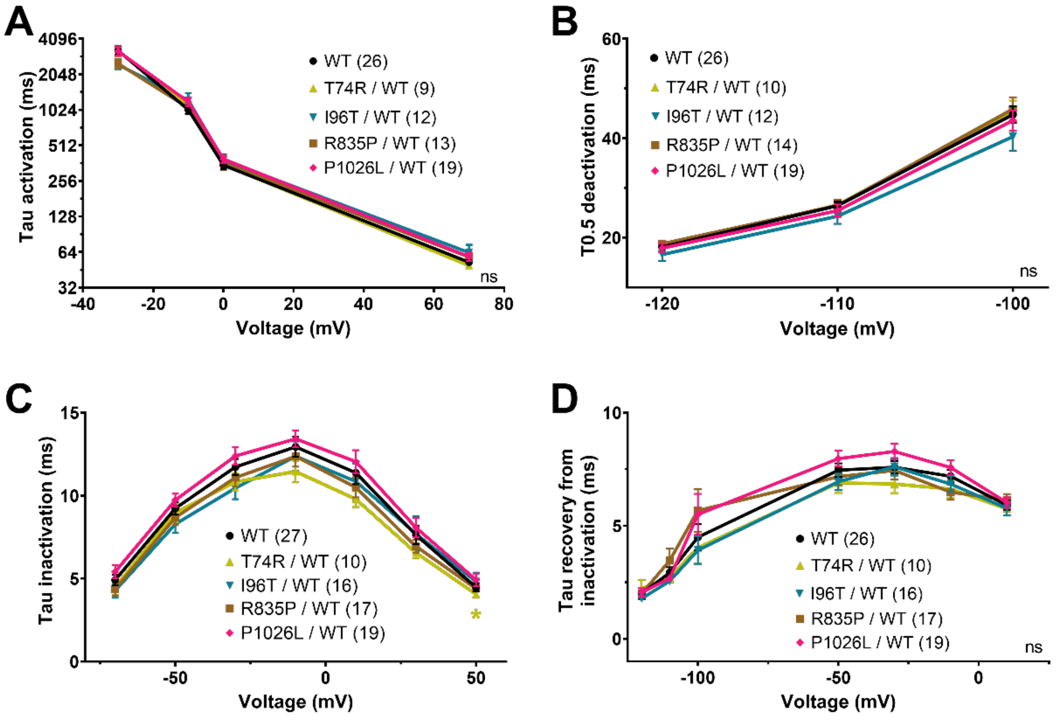

Supplementary Figure S8. **Test of negative dominance on trafficking of four new hERG variants lacking activity in homozygous conditions.** (**A**) Tukey plot of mean ± sem percentage of fluorescence variation (pH 7.4-pH 6.0)/pH 7.4, representing the expression of channels in the cell membrane, corrected by basal pHluorin fluorescence. ****p value vs. WT hERG <0.0001, Kruskal Wallis test. (**B**) and (**C**) Comparison of mean ± sem peak tail hERG current densities from SP3 subprotocols and mean ± sem percentage of fluorescence variation (green). In **C** Colored points represent different hERG variants as in panel **A**. Dotted line is traced from the origin to the WT point to indicate WT current densities vs. fluorescence variation relationship.

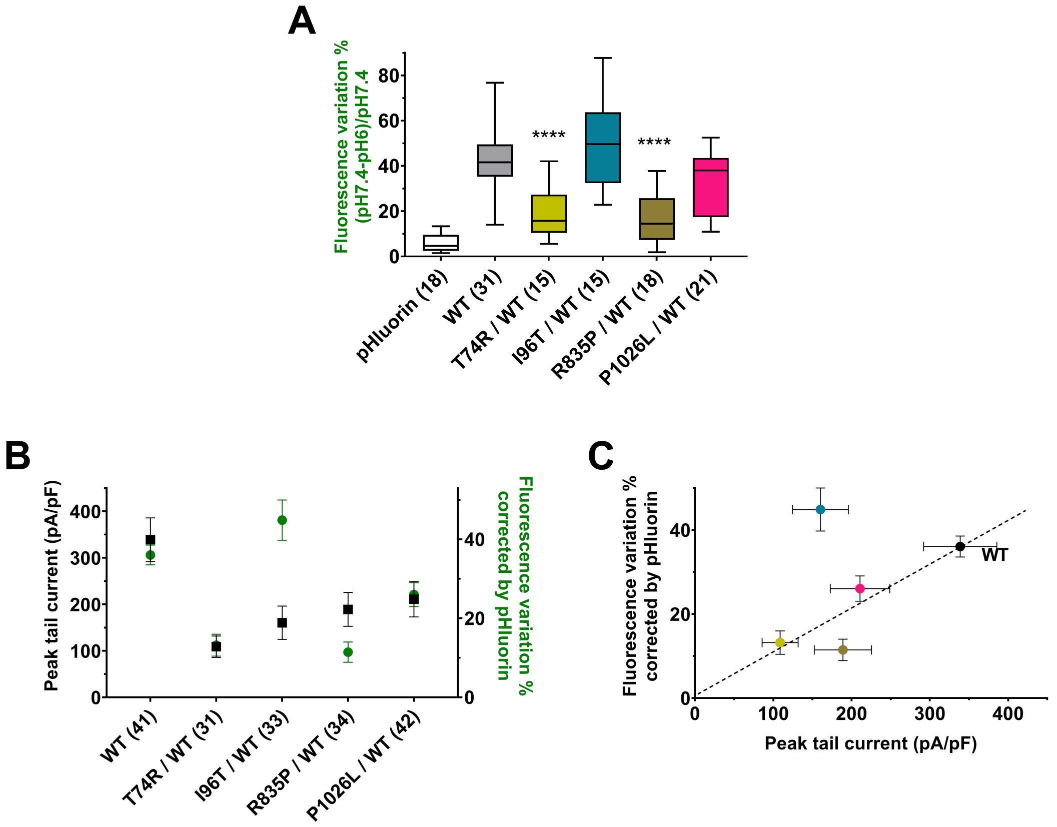

Supplementary Figure S9. **Map of structural impacts, repolarization power and trafficking of hERG variants.** The colored variants indicate the score severity: green - low impact, yellow - medium impact, and red – high impact. **(A)** Map of structural impacts of 10 variants hERG visible in the structure. (**B**) Map of repolarization power impacts of 10 variants hERG visible in the structure. (**C**) Map of trafficking impacts of 10 variants hERG visible in the structure.

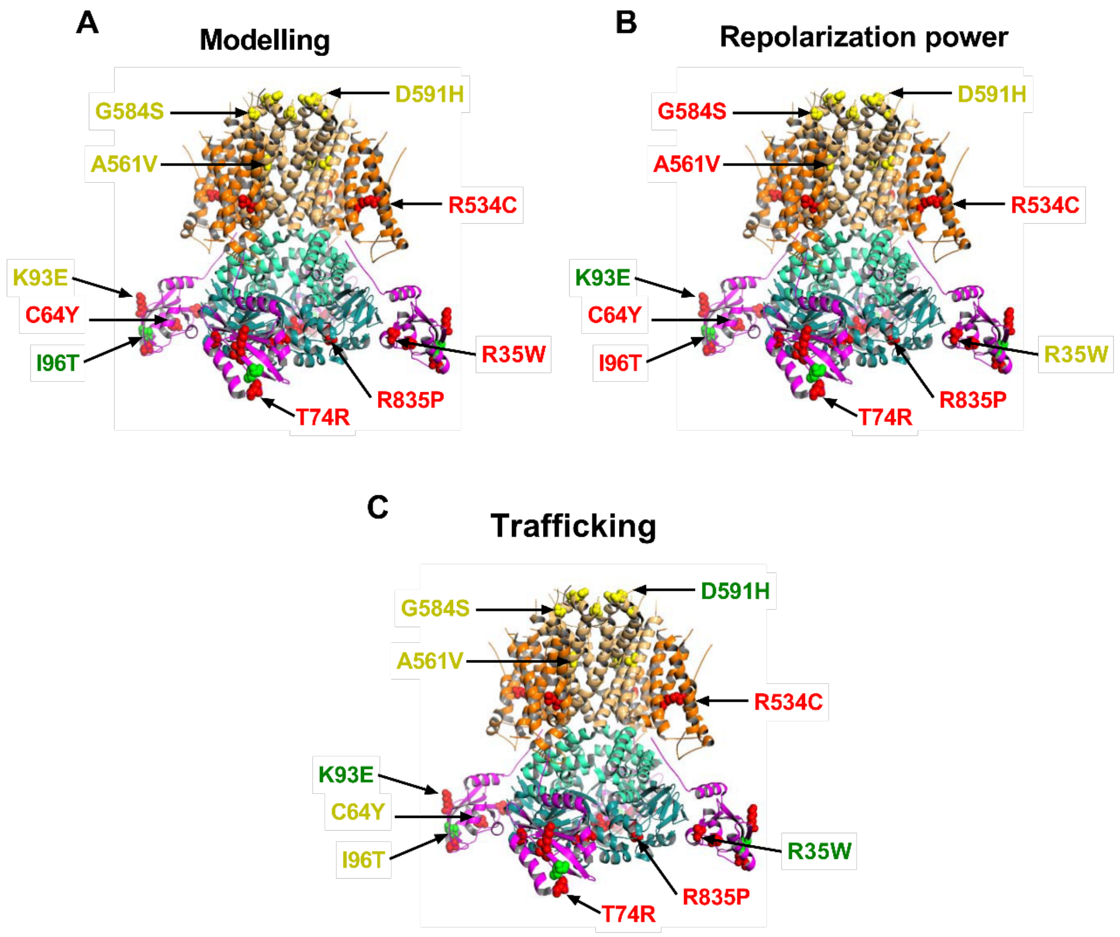

Supplementary text 1 on structural analyses

Regarding criterion A (impact of the mutation on steric hindrance), and assigning a score of 0, 1 or 2 to each mutation, we measured the variations of the side chain occupancy volume for each amino acid substitution. To do this, we calculated, for each possible mutation, the difference between the volume of occupation of the side chain (ΔV) of the new amino acid (Vn) and the volume of that of the original amino acid (Vo) [2]. Since the impact of an increase or a decrease in occupancy volume must be considered with the same attention and interest, we have developed a table displaying for each possible substitution, the absolute values of the differences in occupancy volumes ($V= |Vn-Vo|$) (Top table).

As expected, the largest difference in occupancy volume concerns the substitution of the smallest amino acid (Gly, $V_{Gly} = 0$) by the largest (Trp, $V_{Trp} = 217$ Å^3). In addition, with the introduction of a single methyl group on a residue, the substitution of a Glycine by an Alanine is generally considered negligible from the point of view of steric occupancy (ΔV=67Å^3). We have therefore fixed from 0 to 70 Å^3 the difference for which a change in occupancy volume will have little or no impact (score = 0), from 70 to 140 Å^3 the difference for which the impact will be moderate (score = 1) and beyond 140 Å^3 the difference for which this change in volume has the most important impact (score = 2) The application of this scale to the V Table allowed us to establish the Score Table for criterion A (BottomTable).

	Ala	Arg	Asn	Asp	Cys	Gln	Glu	Gly	His	Ile	Leu	Lys	Met	Phe	Pro	Ser	Thr	Trp	Tyr	Val
Ala	0	129	46	39	37	77	71	67	84	73	70	100	93	108	38	13	35	150	120	50
Arg	129	0	83	90	92	52	58	196	45	56	59	29	36	21	91	116	94	21	9	79
Asn	46	83	0	7	9	31	25	113	38	27	24	54	47	62	8	33	11	104	74	4
Asp	39	90	7	0	2	38	32	106	45	34	31	61	54	69	1	26	4	111	81	11
Cys	37	92	9	2	0	40	34	104	47	36	33	63	56	71	1	24	2	113	83	13
Gln	77	52	31	38	40	0	6	144	7	4	7	23	16	31	39	64	42	73	43	27
Glu	71	58	25	32	34	6	0	138	13	2	1	29	22	37	33	58	36	79	49	21
Gly	67	196	113	106	104	144	138	0	151	140	137	167	160	175	105	80	102	217	187	117
His	84	45	38	45	47	7	13	151	0	11	14	16	9	24	46	71	49	66	36	34
Ile	73	56	27	34	36	4	2	140	11	0	3	27	20	35	35	60	38	77	47	23
Leu	70	59	24	31	33	7	1	137	14	3	0	30	23	38	32	57	35	80	50	20
Lys	100	29	54	61	63	23	29	167	16	27	30	0	7	8	62	87	65	50	20	50
Met	93	36	47	54	56	16	22	160	9	20	23	7	0	15	55	80	58	57	27	43
Phe	108	21	62	69	71	31	37	175	24	35	38	8	15	0	70	95	73	42	12	58
Pro	38	91	8	1	1	39	33	105	46	35	32	62	55	70	0	25	3	112	82	12
Ser	13	116	33	26	24	64	58	80	71	60	57	87	80	95	25	0	22	137	107	37
Thr	35	94	11	4	2	42	36	102	49	38	35	65	58	73	3	22	0	115	85	15
Trp	150	21	104	111	113	73	79	217	66	77	80	50	57	42	112	137	115	0	30	100
Tyr	120	9	74	81	83	43	49	187	36	47	50	20	27	12	82	107	85	30	0	70
Val	50	79	4	11	13	27	21	117	34	23	20	50	43	58	12	37	15	100	70	0

	Ala	Arg	Asn	Asp	Cys	Gln	Glu	Gly	His	Ile	Leu	Lys	Met	Phe	Pro	Ser	Thr	Trp	Tyr	Val
Ala	0	1	0	0	0	1	1	0	1	1	0	1	1	1	0	0	0	2	1	0
Arg	1	0	1	1	1	0	0	2	0	0	0	0	0	0	1	1	1	0	0	1
Asn	0	1	0	0	0	0	0	1	0	0	0	0	0	0	0	0	0	1	1	0
Asp	0	1	0	0	0	0	0	1	0	0	0	0	0	0	0	0	0	1	1	0
Cys	0	1	0	0	0	0	0	1	0	0	0	0	0	1	0	0	0	1	1	0
Gln	1	0	0	0	0	0	0	2	0	0	0	0	0	0	0	0	0	1	0	0
Glu	1	0	0	0	0	0	0	1	0	0	0	0	0	0	0	0	0	1	0	0
Gly	0	2	1	1	1	2	1	0	2	1	1	2	2	2	1	1	1	2	2	1
His	1	0	0	0	0	0	0	2	0	0	0	0	0	0	0	1	0	0	0	0
Ile	1	0	0	0	0	0	0	1	0	0	0	0	0	0	0	0	0	1	0	0
Leu	0	0	0	0	0	0	0	1	0	0	0	0	0	0	0	0	0	1	0	0
Lys	1	0	0	0	0	0	0	2	0	0	0	0	0	0	0	1	0	0	0	0
Met	1	0	0	0	0	0	0	2	0	0	0	0	0	0	0	1	0	0	0	0
Phe	1	0	0	0	1	0	0	2	0	0	0	0	0	0	0	1	1	0	0	0
Pro	0	1	0	0	0	0	0	1	0	0	0	0	0	0	0	0	0	1	1	0
Ser	0	1	0	0	0	0	0	1	1	0	0	1	1	1	0	0	0	1	1	0
Thr	0	1	0	0	0	0	0	1	0	0	0	0	0	1	0	0	0	1	1	0
Trp	2	0	1	1	1	1	1	2	0	1	1	0	0	0	1	1	1	0	0	1
Tyr	1	0	1	1	1	0	0	2	0	0	0	0	0	0	1	1	1	0	0	0
Val	0	1	0	0	0	0	0	1	0	0	0	0	0	0	0	0	0	1	0	0

Top: ΔV Table. *Bottom*: Score Table.
Deducted from Miller S. et al *J. Mol. Biol. 196-:641-656 (1987)*

Concerning criterion B (impact of the change in charge, polarity and affinity of the amino acid for water) and the most convenient way to assign to it a score of 0, 1 or 2, we have elaborated a table that allows to consider at the same time these three parameters for all possible substitutions. To do so, we decided to assign to each mutation a score of:

0 for a substitution that changes neither the charge, nor the polarity nor the hydrophobicity.

1 for a substitution that changes either the polarity or the hydrophobicity or that brings or removes a charge.

2 for a substitution that either changes the polarity or hydrophobicity and brings or removes a charge or changes the sign of the charge of the original amino acid.

While this approach allowed us to easily assign a score to the vast majority of the mutations, we ran into a difficulty that concerns charged residues (Glu, Asp, Arg, Lys and His). These amino acids, and in particular the Histidine residues, present physico-chemical properties that are particularly sensitive to the surrounding conditions, which obliges us to consider the impact of the substitution according to the identity of the amino acids present in their vicinity. Indeed, the presence and the nature of the charge of these residues will depend on both the pH of the surrounding environment and the pKa of this residue. The latter can change depending on the structural context in which it is found in the protein. In order to avoid this difficulty, we have decided to assign these scores according to the substitution without considering the structural factor and to consider the amino acids exchanged individually and at neutral pH. To compensate for the bias introduced by this simplification, we have chosen to integrate, if necessary, a correction index in the C criterion which considers the impact of the mutation on the structure of the protein.

Having taken these different points into consideration, we have established the following table allowing us to assign a score to each mutation.

	Ala	Arg	Asn	Asp	Cys	Gln	Glu	Gly	His	Ile	Leu	Lys	Met	Phe	Pro	Ser	Thr	Trp	Tyr	Val
Ala	0	2	1	2	1	1	2	0	2	0	0	2	0	0	1	1	1	0	1	0
Arg	2	0	1	2	1	1	2	2	1	2	2	0	2	2	1	1	1	2	2	2
Asn	1	1	0	1	0	0	1	1	1	1	1	1	1	1	0	0	0	1	1	1
Asp	2	2	1	0	1	1	0	2	1	2	2	2	2	2	1	1	1	2	2	2
Cys	1	1	0	1	0	0	1	1	1	1	1	1	1	1	0	0	0	1	1	1
Gln	1	1	0	1	0	0	1	1	1	1	1	1	1	1	0	0	0	1	1	1
Glu	2	2	1	0	1	1	0	2	1	2	2	2	2	2	1	1	1	2	2	2
Gly	0	2	1	2	1	1	2	0	2	0	0	2	0	0	1	1	1	0	1	0
His	2	1	1	1	1	1	1	2	0	2	2	1	2	1	1	1	1	1	1	2
Ile	0	2	1	2	1	1	2	0	2	0	0	2	0	0	1	1	1	0	1	0
Leu	0	2	1	2	1	1	2	0	2	0	0	2	0	0	1	1	1	0	1	0
Lys	2	0	1	2	1	1	2	2	1	2	2	0	2	2	1	1	1	2	2	2
Met	0	2	1	2	1	1	2	0	2	0	0	2	0	0	1	1	1	0	1	0
Phe	0	2	1	2	1	1	2	0	1	0	0	2	0	0	1	1	1	0	1	0
Pro	1	1	1	1	0	0	1	1	1	1	1	1	1	1	0	0	0	1	1	1
Ser	1	1	1	1	0	0	1	1	1	1	1	1	1	1	0	0	0	1	1	1
Thr	1	1	1	1	0	0	1	1	1	1	1	1	1	1	0	0	0	1	1	1
Trp	0	2	1	2	1	1	2	0	1	0	0	2	0	0	1	1	1	0	1	1
Tyr	1	2	1	2	1	1	2	1	1	1	1	2	1	1	1	1	1	0	0	0
Val	0	2	1	2	1	1	2	0	2	0	0	2	0	0	1	1	1	0	1	0

Polarity/Hydrophobicity/Charges Table

Supplementary text 2: The full description of all variants hERG structural impacts.

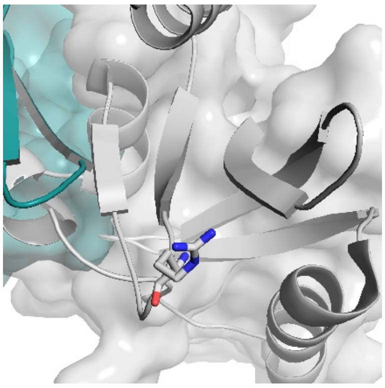

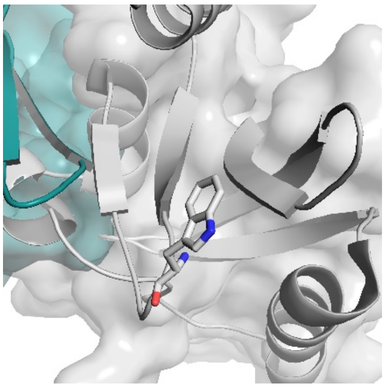

R35W

Arginine #35 belongs to the PAS domain

In the 3D structure available on PDB, this R35 residue resides in a loop region and the sidechain could not be resolved in the cryoEM maps. Its presence in the final structure results from a modeling deduced from a structure probability that considers the amino acids in the vicinity. Thus, the interpretation of the impact of its mutation that is made here is to be considered with the hindsight that is necessary in any model analysis.

If we rely on the position of the residue R35 with respect to the neighboring residues, which are correctly structured, this large hydrophilic positively charged polar amino acid has a strong chance of being in a floating loop and projecting into the cytoplasmic medium. This projection would allow this residue to interact electrostatically with Asp #793 residue located in the CNBHD domain of the neighboring subunit and thus participate in the interaction interface between these two domains. Among the rotamers proposed for this R35 residue, the one with this spatial characteristic is thus chosen. Given the few spatial constraints available to this residue, it can be easily substituted by another residue with similar size, polarity and charge criteria. On the other hand, a hydrophobic aromatic residue such as tryptophan will tend to want to protect itself from the aqueous medium that makes up the cytoplasm and move away from this unfavorable medium by placing itself in the PAS/CNBHD interface, which is more hydrophobic and therefore more favorable to this residue. In doing so, it may reinforce the interaction between these two domains or, on the contrary, because of its size, disturb the geometry of the contact and weaken it. Among the different rotamers proposed in UCSF Chimera, 3 follow this displacement criterion with respective probabilities of 11%, 6% and 5%. The first induces a dramatic clash that cannot be accommodated with residues from the neighboring subunit. The two others induce several non-accommodating clashes with residues from the same subunit. As described in the materials and methods section, if, in the structure minimization calculation of the UCSF Chimera software, a consecutive clash of a mutation cannot be accommodated and compensated by the neighborhood residues then the score that is assigned to this substitution is maximum. Thus, a score of 2 is given

to this third criteria, and a total score of 4 assigned to this variant. Nevertheless, this example with significant reserve introduced by (i) the fact that we work on a modelled part of hERG structure and (ii) the low probabilities assigned to the different rotamers illustrate that this notation should be taken with caution.

A : 0 (similar or close size)

B : 1 + 1 (disappearance of a filler and modification of the hydrophobicity)

C : 2 (appearance of non-accommodating clashes)

Total Score = 4

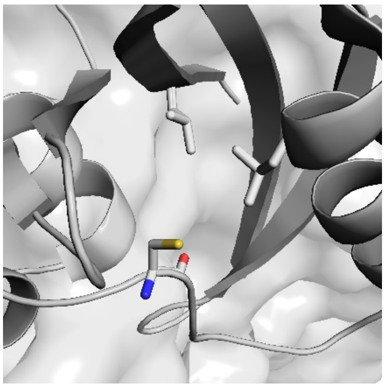
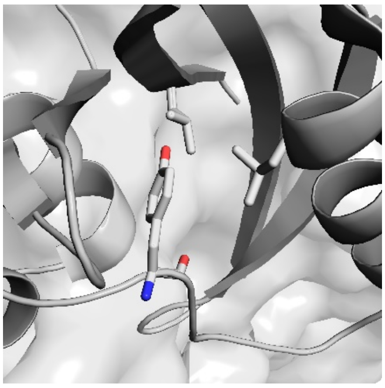

C64Y

Cystein #65 belongs to the PAS domain
Among the 3 most likely rotamers of the C >Y mutation, in position 64, two are in dramatic clash with at least three neighboring residues and one in non-compensable clash with two residues. This mutation will therefore inevitably have an impact on the structure of PAS. The most likely impact concerns a modification of the contact surface between PAS and CNBHD. In the scoring system chosen, this mutation is scored > 4: (A = 1; B = 1; C = 2)
Total Score = 4

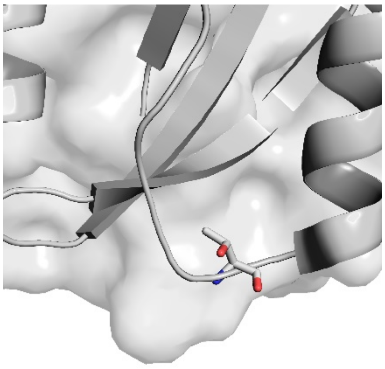
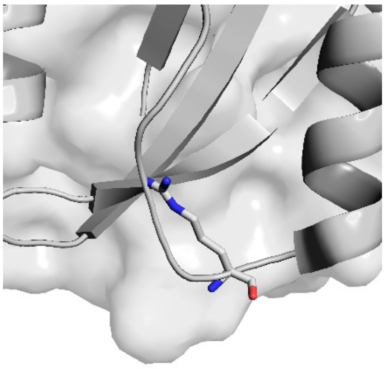

T74R

The Thr74 residue belongs to the PAS domain and projects into a pocket located between the Gβ sheet and the Fα sheet. Ricardo Adaixo et al. [3] showed that in PAS different from hERG, this cavity is important for the binding of regulatory molecules (Flavins for Phot-LOV1 phototropin) and that in hERG, the number of polar residues can be determinant for its ability to detect and bind signals that will modulate its activity.
In the hERG structure, in this pocket, and in the absence of any other molecule, the space is shared between the side chains of the Thr74, His70 and Phe98 residues. Still according to the article cited above, Histidine #70 seems to be a determining factor in the sensor role of this domain.
The substitution of Thr74 by an Arginine will, on the one hand, introduce a moderate spatial constraint (2.3 times the occupied volume, $\Delta V=94$ Å^3) and, on the other hand, introduce positive charges in the vicinity of a histidine. Among the 34 rotamers proposed by the Chimera application, none can accommodate all the clashes listed. The number of clashes before minimization ranges from 38 to a minimum of 11 contacts. This mutation therefore suggests a significant impact on the activity or regulation of the channel.
A : 1
B : 1
C : 2
Total Score = 4

K93E

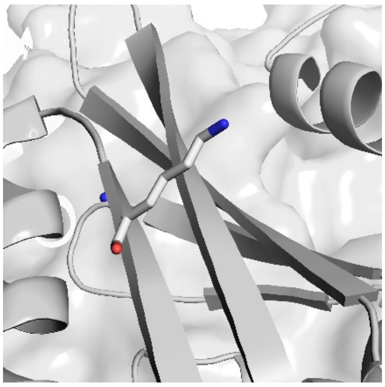

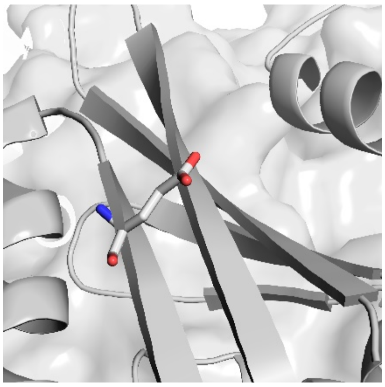

The Lys93 residue is located in the PAS domain. In the crystalline structure, its side chain points towards the extracellular medium and does not seem to interact with other residue of the protein. However, in this same structure, it seems that in this same region should be found a large missing part (part corresponding to the peptide between Val13 and Trp 398, i.e. 267 residues). This does not mean that there is contact between Lysine 93 and this part but it cannot be excluded. Its substitution by a Glutamate will slightly reduce the space occupied by this residue. On the other hand, it will cause a positive charge to disappear and a negative charge to appear, which is (given its position in a critical region of the protein) a major mutation if this residue were to come into contact with another domain of the protein. The search for contact and conflict between this glutamate and other neighboring residues shows an impact in only 10% of the possible rotamers. These contacts are easily compensated by the structure but the movements they imply can be critical for the function of the PAS domain.

A = 0

B = 2

C= 1

Total Score = 3

I96T

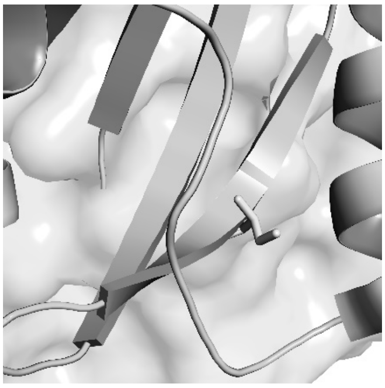

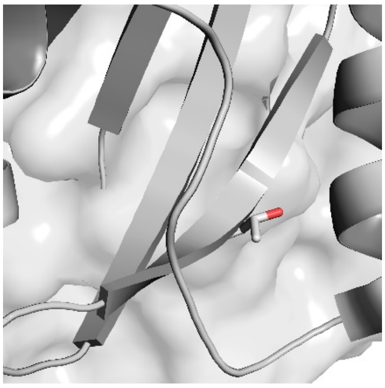

Isoleucine #96 belongs to the PAS domain

Slightly less voluminous than an isoleucine, a threonine introduced at position 96 should not have too much impact from the steric point of view

A : 0 (approximately the same size)

B : 1 The introduced polar grouping is relatively far from the nearest residue (F98 at 3.95Å) and the latter has plenty of room to accommodate a clash if one existed, but no clash with any of the proposed rotamers is demonstrated in the application.

C : 0 (no clash detected in the modeling)

Total Score = 1

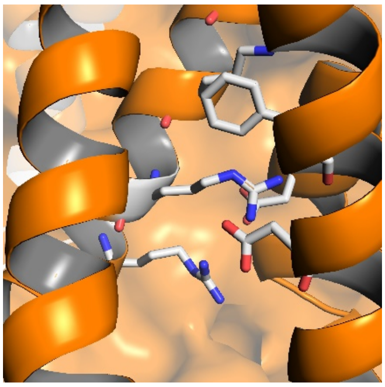

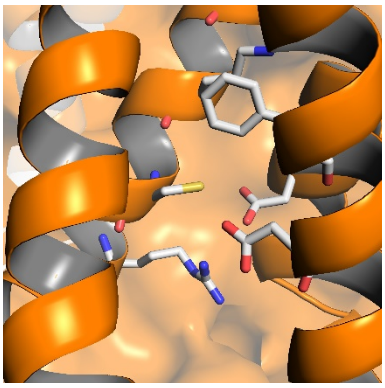

R534C

Arginine #534 belongs to the VSD domain
Located in the S4 domain (voltage sensor) this arginine participates in the cluster of positive charges at the origin of the channel dependent voltage. Its mutation, by any amino acid (other than a Lys) will have an impact on the threshold of the channel activation potential. Given the concomitant change in charge and volume, a total loss of activity cannot be excluded.
A : 1
B : 1
C : 2
Total Score = 4

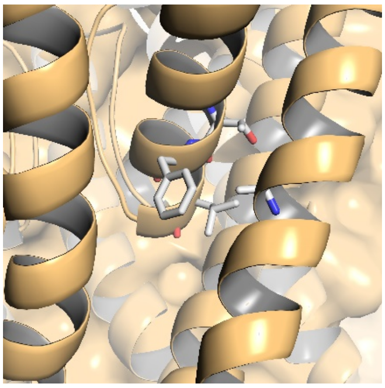

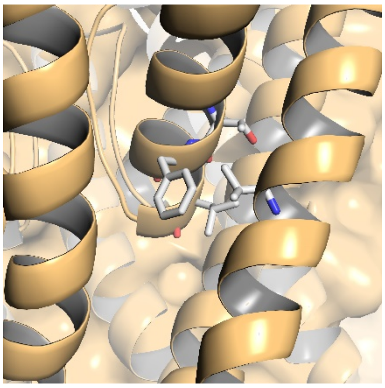

A561V

Alanine #561 belongs to the pore domain
Residue A561 is located in a part of the protein that is very sensitive to changes in geometry and that can indirectly affect the selectivity filter. Its mutation by a radically different amino acid can have dramatic consequences on the activity of the channel. Here, the mutation is relatively conservative and then substitutes a hydrophobic residue by another hydrophobic residue of slightly larger size. It can therefore be assumed that the impact will be small. On the other hand, the orientation of the main rotamers chosen in chimera shows that among the three proposed, only two of them present contacts easily accommodated by the protein (disappearance of 3 clashes and less than 0.3 Å of rotation). The rotamer with the highest probability (92%) is able to compensate only 2 clashes out of the 3 detected by the application. The uncompensated contact is with the F619 residue located on the helix pore whose interactions with the selectivity filter have been shown to be essential in the activation and inactivation mechanisms of the potassium channels.
This mutation is classified as yellow because it requires special attention during electrophysiological verification.
A : 0
B : 0
C : 2
Score Total = 2

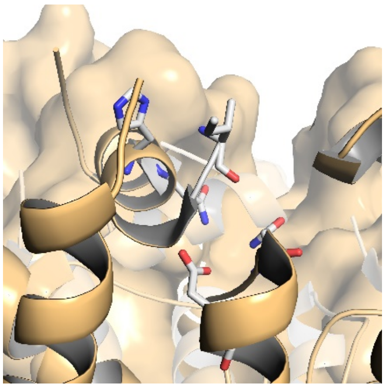

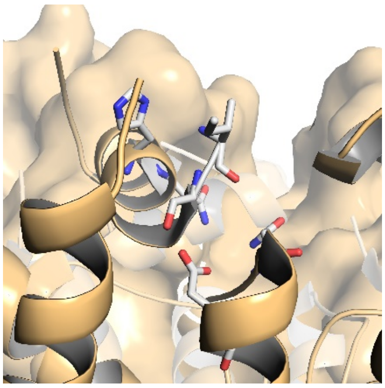

G584S

Glycine #584 belongs to the pore domain (extracellular)
A : 1 (the volume of a Serine is relatively small but still larger than a Glycine)
B : 1 (introduction of a hydroxyl group)
C : 1 the majority rotamer (49% probability) presents 1 clash with Thr #634 very easily minimizable, the secondary rotamer (29% probability) presents 2 clashes (with Thr #634 and Glu #637) relatively easily accommodated, the last rotamer (22% probability) presents 3 clashes with Glu #637 easily accommodated.
This mutation is structurally accommodating but could modify the channel activity because of its position close to the selectivity filter. The question of a prediction of a gain or a loss of function is impossible here.
Total Score = 3

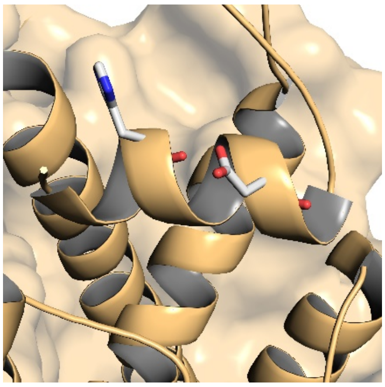

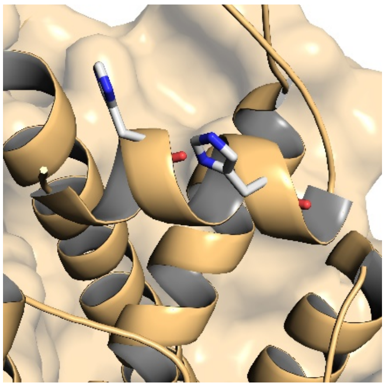

D591H

Aspartate #591 belongs to the pore domain (extracellular)
The radical chain of this residue points into the extracellular space. As such, it is directly exposed to the slightest change in pH of the extracellular medium. Its substitution by Histidine will most likely have no impact on the space occupied (parameter A). Likewise, among the different rotamers proposed by Chimera, only a negligible proportion could induce non-minimizable clashes with neighboring residues (parameter C). On the other hand, the disappearance of the negative charge and its replacement by a protonable residue may be much more critical. To this observation must be considered the sensitivity of this region of the channel to the slightest movement which may affect the geometry of the filter and affect its conductivity by increasing or decreasing it. However, the protonation of a Histidine residue is often associated with a spatial reorganization of the radical chain which can exert physical constraints on the peptide bonds.
This mutation is thus to be considered as potentially critical.
A : 0
B : 1
C : 1
Total Score =2

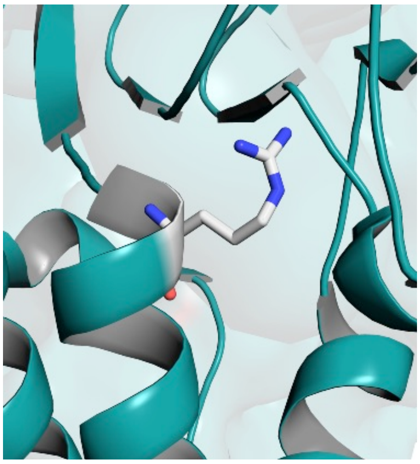

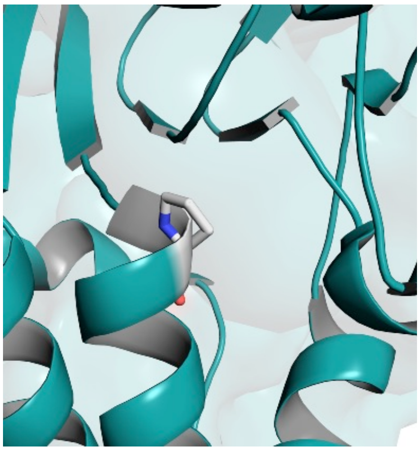

R835P

Arginine #385 belongs to the C-Ter-CNBHD domain. Located at the junction of an alpha helix and a beta leaflet, this basic residue occupies an important place in the architecture of this domain. Its side chain points in the direction of the core of this CNBHD domain and is relatively close in space to the acidic moiety of Residue Glu807 of the same subunit. In the PDB 5VA2 structure, the measured distance between these two groups is 4 Å; a distance too large to sustain a hydrogen bridge but sufficient for an electrostatic interaction. The simulation of the substitution of this Arginine by a Proline shows a significant volume change that moves this proline away from residue E807 and confirms the loss of electrostatic contacts between these two residues. Moreover, and considering the position of this Arginine in the helix a mentioned above, an important modification of the structure is to be envisaged during this mutation. Indeed, the "Helix Breaker" character of the proline can presage a significant modification of the geometry of the helix and thus of any interactions that this residue may have with its neighborhood. We therefore assign a score of 2 for criterion C. In addition, the change in size and charge adds to the overall score. Based on the score tables for criteria A and B (size and hydrophobicity), each of these will contribute one score point.

A =1

B= 1

C= 2

Total Score = 4

References

1. O'Hara T, Virag L, Varro A, Rudy Y. Simulation of the undiseased human cardiac ventricular action potential: Model formulation and experimental validation. *PLoS Comput Biol*. 2011;7:e1002061
2. Miller S, Janin J, Lesk AM, Chothia C. Interior and surface of monomeric proteins. *J Mol Biol*. 1987;196:641-656
3. Adaixo R, Harley CA, Castro-Rodrigues AF, Morais-Cabral JH. Structural properties of pas domains from the kcnh potassium channels. *PLoS One*. 2013;8:e59265
